# Signal peptide of HIV-1 envelope modulates glycosylation impacting exposure of V1V2 and other epitopes

**DOI:** 10.1371/journal.ppat.1009185

**Published:** 2020-12-28

**Authors:** Chitra Upadhyay, Roya Feyznezhad, Liwei Cao, Kun-Wei Chan, Kevin Liu, Weiming Yang, Hui Zhang, Jason Yolitz, James Arthos, Arthur Nadas, Xiang-Peng Kong, Susan Zolla-Pazner, Catarina E. Hioe

**Affiliations:** 1 Icahn School of Medicine at Mount Sinai, New York, New York, United States of America; 2 James J. Peters Veterans Affairs Medical Center, Research Service, Bronx, New York, United States of America; 3 Johns Hopkins University, Baltimore, Maryland, United States of America; 4 Department of Biochemistry and Molecular Pharmacology New York University School of Medicine, New York, New York, United States of America; 5 Laboratory of Immunoregulation, National Institute of Allergy and Infectious Diseases, National Institutes of Health, Bethesda, Maryland, United States of America; 6 Institute of Environmental Medicine, New York University School of Medicine, New York, New York, United States of America; University of Zurich, SWITZERLAND

## Abstract

HIV-1 envelope (Env) is a trimer of gp120-gp41 heterodimers, synthesized from a precursor gp160 that contains an ER-targeting signal peptide (SP) at its amino-terminus. Each trimer is swathed by ~90 N-linked glycans, comprising complex-type and oligomannose-type glycans, which play an important role in determining virus sensitivity to neutralizing antibodies. We previously examined the effects of single point SP mutations on Env properties and functions. Here, we aimed to understand the impact of the SP diversity on glycosylation of virus-derived Env and virus neutralization by swapping SPs. Analyses of site-specific glycans revealed that SP swapping altered Env glycan content and occupancy on multiple N-linked glycosites, including conserved N156 and N160 glycans in the V1V2 region at the Env trimer apex and N88 at the trimer base. Virus neutralization was also affected, especially by antibodies against V1V2, V3, and gp41. Likewise, SP swaps affected the recognition of soluble and cell-associated Env by antibodies targeting distinct V1V2 configurations, V3 crown, and gp41 epitopes. These data highlight the contribution of SP sequence diversity in shaping the Env glycan content and its impact on the configuration and accessibility of V1V2 and other Env epitopes.

## Author summary

HIV-1 Env glycoprotein is produced by a precursor gp160 that has a signal peptide at its N-terminus. The SP is highly diverse among the HIV-1 isolates. This study presents site-specific analyses of N-linked glycosylation on HIV-1 envelope glycoproteins from infectious viruses produced with different envelope signal peptides. We show that signal peptide swapping alters the envelope glycan shield, including the conserved N156 and N160 glycans located in the V1V2 region on the trimer apex, to impact Env recognition and virus neutralization by antibodies. The data offer crucial insights into the role of signal peptide in the interplay between HIV-1 and antibodies and its potential utility to control Env glycosylation in the development of Env-based HIV-1 vaccine.

## Introduction

The HIV-1 envelope glycoprotein (Env), the only viral protein accessible to neutralizing antibodies, is a critical HIV-1 vaccine component. HIV-1 Env is synthesized as a precursor gp160 glycoprotein, which is directed to the endoplasmic reticulum (ER) by its 30-amino-acid N-terminal signal peptide (SP, also known as signal or leader sequence). In general, SPs contain 16 to 30 amino acids with a characteristic tripartite structure: a hydrophilic positively charged n-region, a central hydrophobic h-region, and a slightly polar carboxy terminal c-region with a cleavage site for signal peptidase [1]. In the ER, gp160 is decorated with ∼30 N-linked glycans and undergoes folding and extensive isomerization until near native conformation is reached with the SP still attached. The SP is cleaved before gp160 reaches the Golgi for further glycosylation maturation and other post-translational modifications [2–4]. In the Golgi, gp160 is cleaved by host protease furin to generate transmembrane gp41 subunit and noncovalently associated surface gp120 subunit; three gp120-gp41 heterodimers assemble to form the functional Env spikes. The apex of the trimeric spike is made of V1V2 domains from the three gp120 protomers with V3 loops laying underneath and partially concealed in the native “closed” conformation [5–7]. V1V2 epitopes are of particular interest, since the RV144 Thai vaccine trial identified high levels of V1V2-specific antibodies as a correlate of reduced infection risk [8–10]. While V1V2 and V3 contain cross-reactive immunogenic epitopes, these regions are structurally dynamic and the epitopes are conformationally masked in most HIV-1 isolates [11]. Dense glycosylation further shields the V1V2, V3 and other epitopes [12–14]. Nonetheless, some glycans also serve as components of epitopes for broadly neutralizing antibodies (bNAbs) [15–18].

Each V1V2 domain can form a 5-strand beta-barrel module, with the polymorphic V2 C-strand adopting α-helical or β-sheet conformations [19]. Three types of V1V2 epitopes have been defined: V2p, V2i and V2q [20]. The V2p type constitutes the α-helical peptide epitopes in the V2 C-strand (V2C) that are recognized by monoclonal antibodies (mAbs) CH58 and CH59 from RV144 vaccinees [21] and the CAP228 mAb series from a virus-infected donor in South Africa [22]. The V2i-type epitopes are made of discontinuous segments in V1V2, encompassing the α4β7 integrin-binding motif [23,24]; these epitopes are recognized by mAbs such as 830A, 697-30D, and 2158 isolated from infected US patients [23]. The V2q type is comprised of quaternary neutralizing epitopes targeted by bNAbs such as PG9 and PG16 [25]. The V2q epitopes require that V2C assume a beta-sheet structure [16]. The V2q epitopes also include glycans at positions N156 or N173 and N160 on the V1V2 apex [16,26], which together with N197 and N276 glycans create the conserved ‘trimer-associated mannose patch’ (TAMP). The V2i epitopes, in contrast, do not include glycans, but are conformationally dependent on proper V1V2 glycosylation and folding, and require a beta-strand V2C configuration [19,20]. The propensity of V2C to adopt α-helical and β-sheet structures is influenced by glycosylation [20]. Thus, changes in glycosylation can impact the interaction of Abs with V1V2 and other Env regions as well.

We and others have shown that SP can alter HIV-1 Env glycosylation [27,28]. Expressing HIV-1 gp120 with SPs from heterologous isolates also altered gp120 glycosylation and reactivity with lectins and mAbs [28]. The HIV-1 SPs display remarkable sequence diversity [27,29,30], the reason for which are not fully understood. Nonetheless, the native Env SP has been replaced by unrelated SPs (e.g. SPs of CD5 and tissue plasminogen activator (tPA)) to promote the secretion of soluble Env [30–33]. Likewise, herpes simplex virus (HSV) SP was used to express gp120 tested in RV144 vaccine trial [34–36], the only phase IIb/III trial with a modest 31 % protective efficacy [34–36]. SPs are typically cleaved from the maturing protein co-translationally, but the SP of HIV-1 Env is cleaved post-translationally [3,4,31,37]. This delayed SP cleavage is conserved across HIV-1 subtypes and functions as a quality-control checkpoint, ensuring proper folding and post-translational modifications of gp160 before its egress from the ER [38]. Indeed, beyond protein targeting, SP influences protein folding, modification, localization, and topology [37,39–41]. Mutations in SPs are suggested to impair transport and alter maturation as a result of incomplete glycosylation in the ER and Golgi, leading to or associated with several human diseases [42–47]. The effect of SP and its residues on viruses has not been much studied. The extremely long Env SPs of retroviruses like foamy virus and mouse mammary tumor virus have been shown to mediate additional functions other than ER targeting. The cleaved SP product from foamy virus is associated with cell-free virions and is critical for virus release from infected cells [48]. The SP of the MMTV envelope precursor is reported to function as a nuclear export factor for intron-containing transcripts [49]. Collectively, these studies highlight the critical roles of SPs and their profound effects on the functions of associated proteins and viruses.

**Fig 1:**
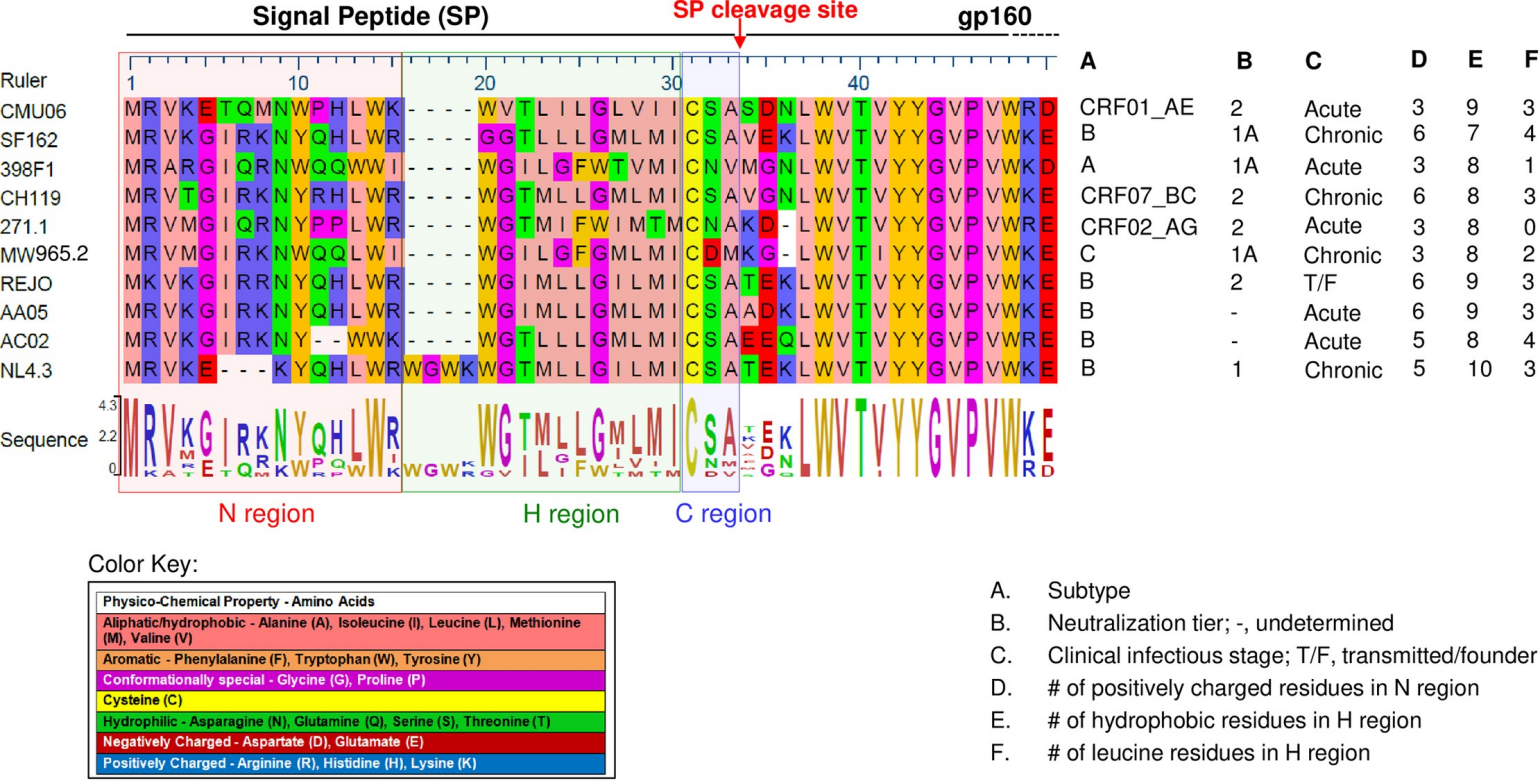
Sequence alignment of all Env SPs tested in this study. Amino acid sequence alignment was performed using the Lasergene DNASTAR Megalign software. Dashes (-) indicate missing residues.

We previously reported that the HIV-1 Env glycan content can be altered by single point mutations in the Env SP to affect virus sensitivity to neutralization by antibodies; the glycan alterations were apparent in lectin-probed Western blots and by LC-MS/MS, although only two of 29 potential N-linked glycosites (PNGSs) were detected by LC-MS/MS [27]. In this study we identified an array of PNGSs impacted by exchanging the entire native SP with SPs from heterologous HIV-1 isolates in the proviral context. Using Env backbones and SPs from viruses representing different clades, neutralization tiers and clinical stages (Fig 1), we tested the impact of SP exchanges on Env functions in the setting of different Env backbones. Site-specific glycosylation analysis of virion-derived total Env expressed with SPs from heterologous HIV-1 strains revealed altered proportions of oligomannose and complex glycans at many glycosites including those at the trimer apex and base. The modified glycan compositions were associated with perturbed virus neutralization and Env binding by mAbs targeting different Env epitopes, including mAbs specific for V1V2, V3 and gp41. As SP swapping is a common strategy employed in constructing Env-based HIV-1 vaccines, this study provides previously unknown details about the SP impact on HIV-1 Env properties with important implications for vaccine designs.

## Results

### Glycosylation changes of SP-swapped vs WT Envs on CMU06 virions

**Fig 2:**
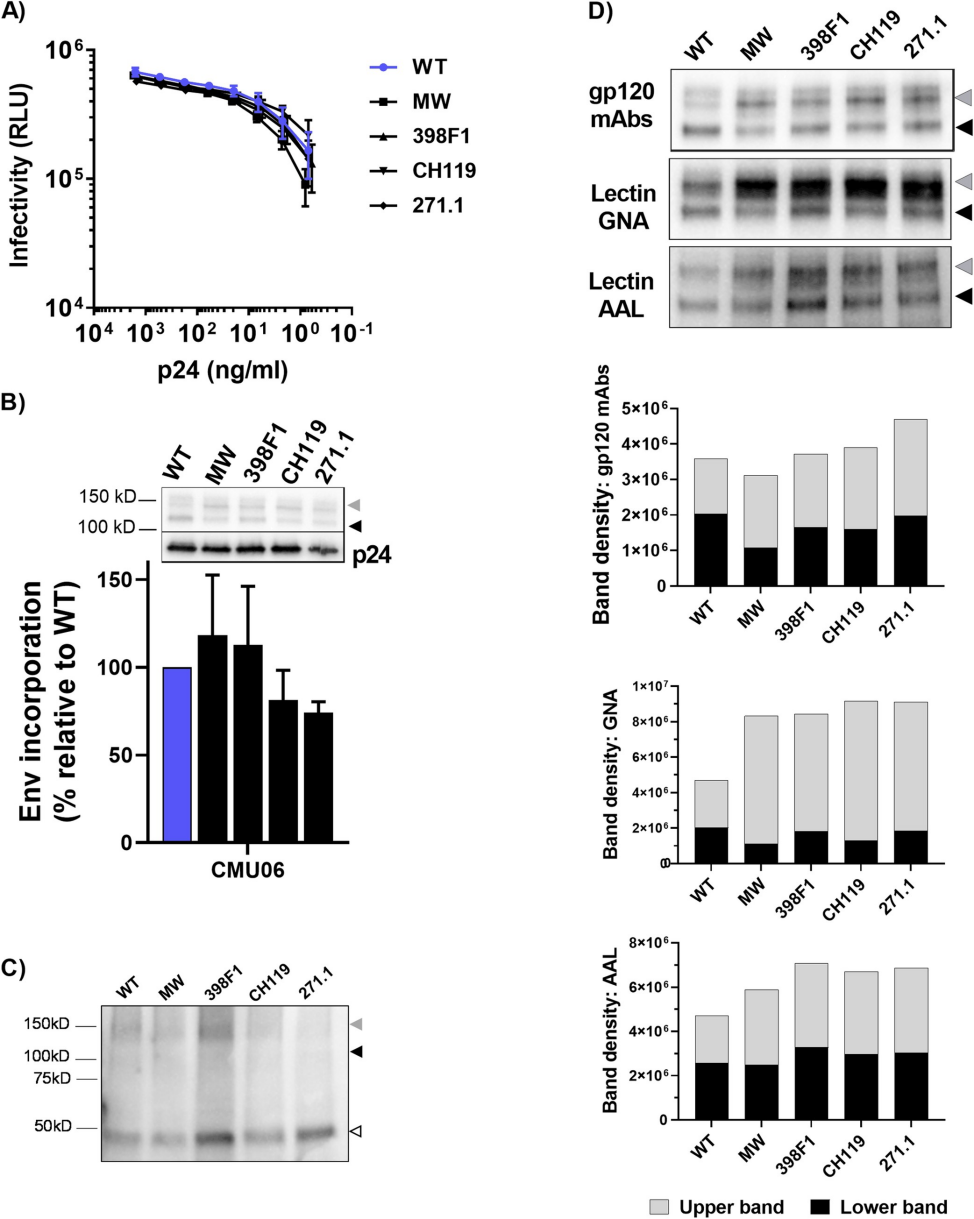
Effect of SP swap on CMU06 infectivity, Env incorporation and lectin binding. (A) Infectivity of 293T-derived SP-swapped vs WT CMU06 viruses in TZM.bl cells. (B) Measurement of Env incorporation by Western blots. (C) Western blot probed with gp41 MPER mAb 2F5. (D) Binding of Env from CMU06 WT and SP-swapped viruses by gp120 mAbs and lectins (GNA and AAL) in Western blots. Upper gp160, lower gp120, and gp41 Env bands are denoted by gray, black, and white triangles, respectively.

To investigate the functional consequences of Env SP diversity, we selected SPs from isolates that display different neutralization sensitivity and represent different clades and clinical stages [30,50,51]. The SP of CMU06 Env (CRF01_AE, tier 2, acute) was swapped with SPs from MW965.26 (clade C, tier 1A, chronic), 398F1 (clade A, tier 1A, acute), CH119 (CRF_07, tier 2, chronic) and 271.1 (clade C, tier 2, acute), while maintaining the SP cleavage site of the parental CMU06 (Fig 1). To determine the effect of SP swap on infectivity, we generated viruses by transfecting 293T cells with infectious molecular clone (IMC) of CMU06 with WT and SP-swapped Envs. The infectivity of all SP swapped viruses was comparable to the WT (Fig 2A). The ratios of Env and Gag p24 incorporated into virions were also similar, as indicated by Western blot with anti-gp120 and anti-p24 mAbs (Fig 2B). Upper gp160 and lower gp120 bands were detected by anti-gp120 mAbs. The ratios of total Env/p24 were calculated for each virus as the percentage of WT. The blot was also probed with anti-gp41 MAb 2F5, which detected gp41 and gp160 but not gp120 (Fig 2C).

To appraise the impact of SP swapping on glycosylation, we first tested Envs from sucrose-pelleted CMU06 viruses in Western blots probed with anti-gp120 mAbs versus lectins that recognize distinct sugar moieties: GNA (terminal α1–3 and α1–6 mannose on high-mannose glycans) and AAL (fucose on complex glycans) [52,53] (Fig 2D). GNA reactivity to total Envs of SP-swapped viruses increased by ~2 fold vs WT, whereas anti-gp120 binding did not increase to the same level. Notably, GNA reacted more strongly to the upper band of SP-swapped Envs, while binding to the lower bands was minimally altered (Fig 2D). Differences were also observed in AAL binding to the upper and lower bands of all SP-swapped Envs. These data demonstrated modulations in glycan contents of SP-swapped Envs vs WT that were modest but enriched for terminal α1–3 and α1–6 mannose-bearing oligomannose-type glycans and fucosylated complex glycans.

To further discern glycan alterations at specific N-linked glycosites, sucrose-purified CMU06-WT, CMU06-398F1, and CMU06-271.1 were subjected to liquid chromatography–mass spectrometry (LC-MS/MS) upon Endo-H and PNGase digestion. HIV-1 virions are known to carry both functional and non-functional Envs in different conformations [54], and different glycoforms may exist on these varied Env forms. While functional Env spikes are required for virus infectivity, the non-functional forms of Env may serve as decoys to avert antibody recognition detrimental to the virus [55]. In this study, we were not able to separate the different Env forms with sufficient purity and quantity. We also did not perform affinity purification with lectins or antibodies to avoid enriching for particular Env forms. Thus, LC-MS/MS analyses were conducted on total Env from both upper and lower Env bands detected on SDS-PAGE of concentrated virus preparations.

**Fig 3:**
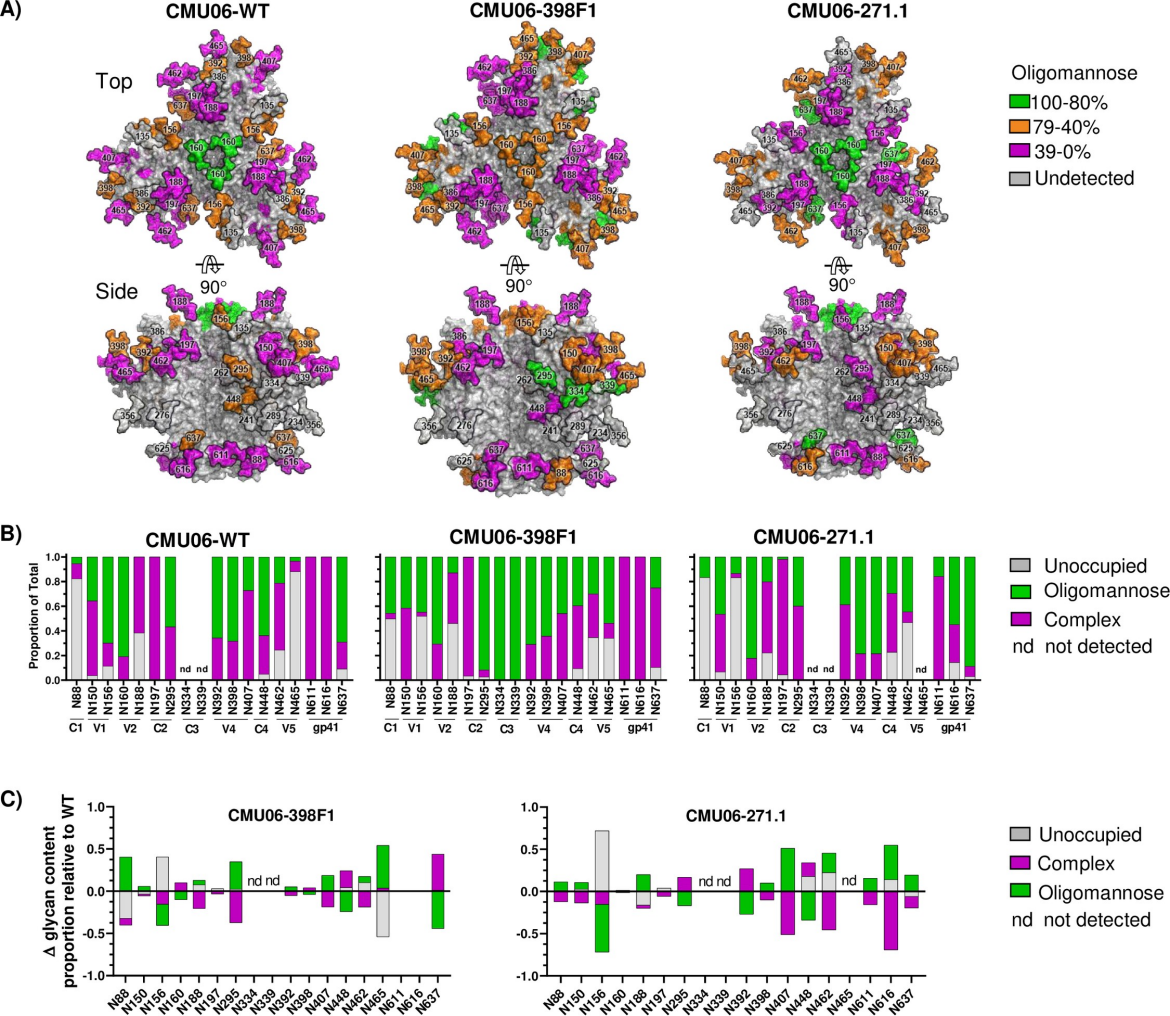
Effect of SP swap on CMU06 Env glycosylation. (A) Env trimer models (generated using a SOSIP.664 trimer PDB 5FYJ) showing the predominating glycoforms at each of the CMU06 glycosites for CMU06-WT, CMU06-398F1, and CMU06-271.1. Type of glycans added was based on LC-MS/MS data of virion-associated Envs from 293T cells (Fig 3B). (B) Relative amounts of unoccupied, oligomannose, and complex glycans at glycosites on SP-swapped vs WT CMU06 as determined by LC-MS/MS. nd: glycosites not detected in 1 or 2 Envs. Glycosites undetected in all 3 Envs are not shown. (C) Changes in glycan content at each glycosite for CMU06-398F1 and CMU06-271.1 vs CMU06-WT calculated from Fig 3B data.

Of 28 potential glycosites present on CMU06 Env (S1 Fig), oligomannose glycans, complex glycans, and unoccupied glycosites were identified with coverage of 57% (16/28), 64% (18/28), and 54% (15/28) respectively. Hybrid-type glycans were indistinguishable from oligomannose-type, as both were digested by Endo-H. Noticeable alterations were found at many glycosites in gp120 and gp41 of CMU06-398F1 and CMU06-271.1 vs CMU06-WT (Fig 3A–3C). Of the 12 gp120 glycosites detected on all 3 Envs, the percentage of oligomannose glycans was same on CMU06-WT (41%) and CMU06-271.1 (41%) but changed to 47% on CMU06-398F1. Of the three gp41 glycans, complex types were predominant on WT (74%) and the amounts were increased on 398F1 (88%) and decreased on 271.1 (41%). As a whole, LC-MS/MS data demonstrated an increase in total oligomannose glycans of CMU06-398F1 (40%) and CMU06-271.1 (43%) vs CMU06-WT (35%), consistent with GNA binding data (Fig 2D). The data also showed alterations in the total complex glycans (45%: 398F1 and 38%: 271.1 vs 48%: WT), but LC-MS/MS could not delineate the specific proportion of fucose-bearing glycans detected by AAL.

A model of glycosylated trimeric Env was generated to illustrate glycosite-specific alterations on CMU06-WT and SP swapped viruses (Fig 3A). Changes were observed in glycan contents of N156 and N160 at the trimer apex (Fig 3A–3C). N156 and N160 glycans constitute the TAMP and serve as key contact residues for bNAbs such as PG9, PG16, PGT145 and PGDM1400 [16,56,57]. On WT, N156 was occupied by oligomannose (70%) and complex (19%) glycans, while 11% was unoccupied. In contrast, N156 was largely unoccupied on CMU06-398F1 (52%) and CMU06-271.1 (83%). When N156 of CMU06-398F1 was occupied, oligomannose glycans predominated. N173, which is recognized in place of N156 by PG9 on some HIV-1 isolates [58], is absent on CMU06 (S1 Fig). N160 was decorated mostly by oligomannose glycans in CMU06-WT and CMU06-271.1 (81–82%) but had lower abundance in CMU06-386F1 (71%). Complex-type glycans increased proportionally to 29% at N160 of CMU06-386F1 vs 19% in CMU06-WT and 18% in CMU06-271.1. At nearby N188, also emanating from the apical V1V2 region, 398F1 and 271.1 SP swapping increased oligomannose glycans to 13% and 20%, respectively, from 0% in CMU06-WT.

Additional changes were also noted in glycans projecting laterally from the Env core at positions N295, N392, N407, N448, N462, and N465. N295 and N392, which together with N332 or N334 comprise the intrinsic mannose patch (IMP), had lower or higher proportions of complex glycans in SP-swapped viruses vs WT, whereas N334 was detected in only CMU06-398F1 to contain oligomannose glycans. However, not all glycosites were affected by SP swapping; minimal to no changes were observed at N197, 94–100% of which was comprised of complex glycans, and at N398, which contained complex and oligomannose glycans at a ratio of ~1:2 in all three Envs tested.

The N88 glycan at the trimer base, which shields the MPER epitope [59], was largely unoccupied in CMU06-WT (82%) and CMU06-271.1 (82%) but had 50% occupancy in CMU06-398F1 with mainly oligomannose glycans. N611 and N616 at the trimer base in gp41 were 100% occupied by complex glycans in CMU06-WT and CMU06-398F1, but in CMU-271.1 oligomannose glycans were found at N611 (16%) and N616 (55%). The oligomannose content of N637 in gp41 increased from 69% in CMU06-WT to 89% in CMU06-271.1 but was reduced to 25% in CMU06-398F1. These glycans are among those on the gp120-gp41 interface essential for Env function [60] and constituting epitopes of PGT151, 35O22 and 8ANC195 bNAbs [60–63]. Glycosites at N135, N234, N241, N262, N276, N289, N356, N386 and N625 were undetectable in all three Envs. Collectively, the data provides insights into the role of SP in influencing glycan composition at specific Env glycosites.

### Altered mAb recognition of CMU06 as a result of SP swapping

**Fig 4:**
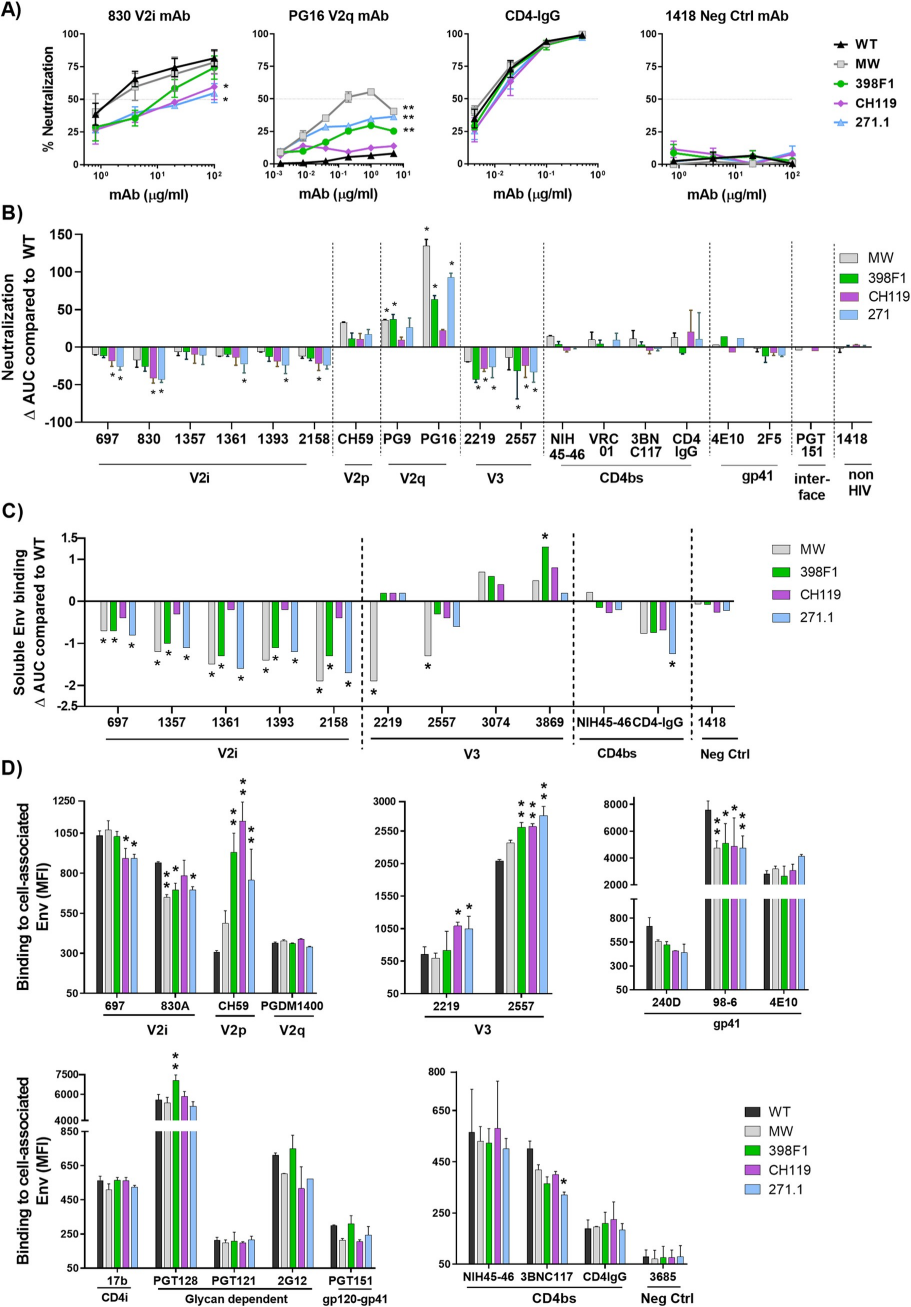
Effect of SP swap on CMU06 Env-antibody interaction. (A) Neutralization of 293T-derived SP-swapped vs WT CMU06. Viruses were incubated with titrated mAbs at 37°C for 24 hours before addition of TZM.bl cells, except for PG9, NIH45-46, VRC01, 3BNC117, 4E10, 2F5, PGT151 and CD4IgG which were pre-incubated with viruses for 1 hour. Anti-parvovirus mAb 1418 served as negative control. Representative neutralization curves are shown. Mean and SEM from 2–3 experiments are shown. Data were analyzed by 2-way ANOVA (* p < 0.05, ** p < 0.01, vs WT). (B) Changes in neutralization AUCs (areas under the curves) of SP-swapped vs WT CMU06 with different mAbs. AUC changes are calculated as follows: (SP-swapped AUC–WT AUC). Statistical analysis was performed on neutralization curves (Fig 4A) by 2-way ANOVA (* p < 0.05) (C) ELISA binding of mAbs to solublized Env derived from CMU06-WT vs SP-swapped viruses. Virus-derived Env was captured by sheep anti-C5 antibody (1μg/ml) and reacted with different mAbs titrated ten-fold from 10 μg/ml. CD4IgG was titrated five-fold from 10 μg/ml. AUC values were calculated from binding curves. AUC changes relative to the respective WT are shown. Statistical analysis was performed on binding curves by 2-way ANOVA (* p < 0.05) (D) Binding of mAbs to WT vs SP-swapped Envs expressed on the surface of 293T cells. MAbs were tested at following concentrations: 697, 830 and 3685 at 100μg/ml; 2G12 at 50μg/ml; PGDM1400, 2219, 2557, 240D, 98–6 and PGT121 at 25μg/ml; b12 at 20μg/ml; 4E10, 17b at 10μg/ml; PGT128, PGT151, 3BNC117 and CD4IgG at 5μg/ml; CH59 and NIH45-46 at 2.5μg/ml. Geometric mean fluorescence intensity (MFI) and SD from duplicates in one experiment are shown. Data were analyzed by one-way ANOVA (* p < 0.05, ** p < 0.01, vs WT).

Next, we examined the effect of SP swap on virus neutralization. Changes in sensitivity to neutralization were observed with each SP-swapped virus vs WT (Fig 4A and 4B). CMU06 viruses with CH119 and 271.1 SP were more resistant to neutralization by most V2i mAbs tested. Increased sensitivity to V2q mAbs PG9 and/or PG16 was observed for viruses with SPs from MW965.26, 398F1, and 271.1. Neutralization of CMU06-WT by V3 crown-specific mAbs 2219 and 2557 was weak, but switching SPs further reduced neutralization by 2219 and 2557. Neutralization by V2p mAb (CH59), gp41-MPER mAb (2F5 and 4E10), CD4bs mAbs and CD4IgG was not much affected (Fig 4A and 4B). No neutralization was observed by the gp120-gp41 interface mAb PGT151 (IC50 >20μg/ml), and no change was incurred with SP swapping.

To evaluate the impact of SP swapping on antibody binding, we tested mAb binding to virus-derived solubilized Env captured onto ELISA plates and native Env expressed on the surface of 293T cells. ELISA with equivalent amounts of Env showed reduced binding of all V2i mAbs tested to Env from CMU06-MW965.26, CMU06-398F1, and CMU06-271.1, whereas binding to CMU06-CH119 was less affected (Fig 4C). Considering that the viruses had an identical V1V2 sequence, the data indicate that SP swapping induced alterations of the V2i structure and/or exposure to reduce mAb recognition. Sporadic changes were also seen with V3 mAbs and CD4IgG, while binding to CD4bs mAb NIH45-46 was not much affected.

Next, we tested if SP swapping triggered antigenic changes on Envs expressed on the 293T cell surface. CD4IgG had comparable binding, indicating equivalent expression of all five Envs. Notably, differences were observed with V2i and V2p mAbs (Fig 4D). V2i mAb 830 had reduced binding to three of the four SP swaps vs CMU06-WT. Binding of V2i mAb 697 for CMU06-CH119 and CMU06-271.1 was also reduced. V2p mAb CH59 had greater binding to three of the SP swaps vs CMU06-WT. Binding of V3 crown mAbs 2219 and 2557 was increased to two and three SP swaps, respectively. The gp41-specific mAb (98–6) had reduced reactivity with all four SP-swaps vs WT. In contrast, binding of V2q mAb PGDM1400, CD4i mAb 17b, glycan-dependent mAb PGT121 and 2G12, gp120-gp41 interface mAb PGT151, CD4bs mAb NIH45-46, and gp41 mAbs 240D and 4E10 to SP-swapped and WT Envs was comparable. These data demonstrated that SP swapping altered Env antigenicity impacting V1V2, V3 and gp41 epitopes.

### Effect of SP swapping on REJO Env incorporation, glycosylation and antibody recognition

**Fig 5:**
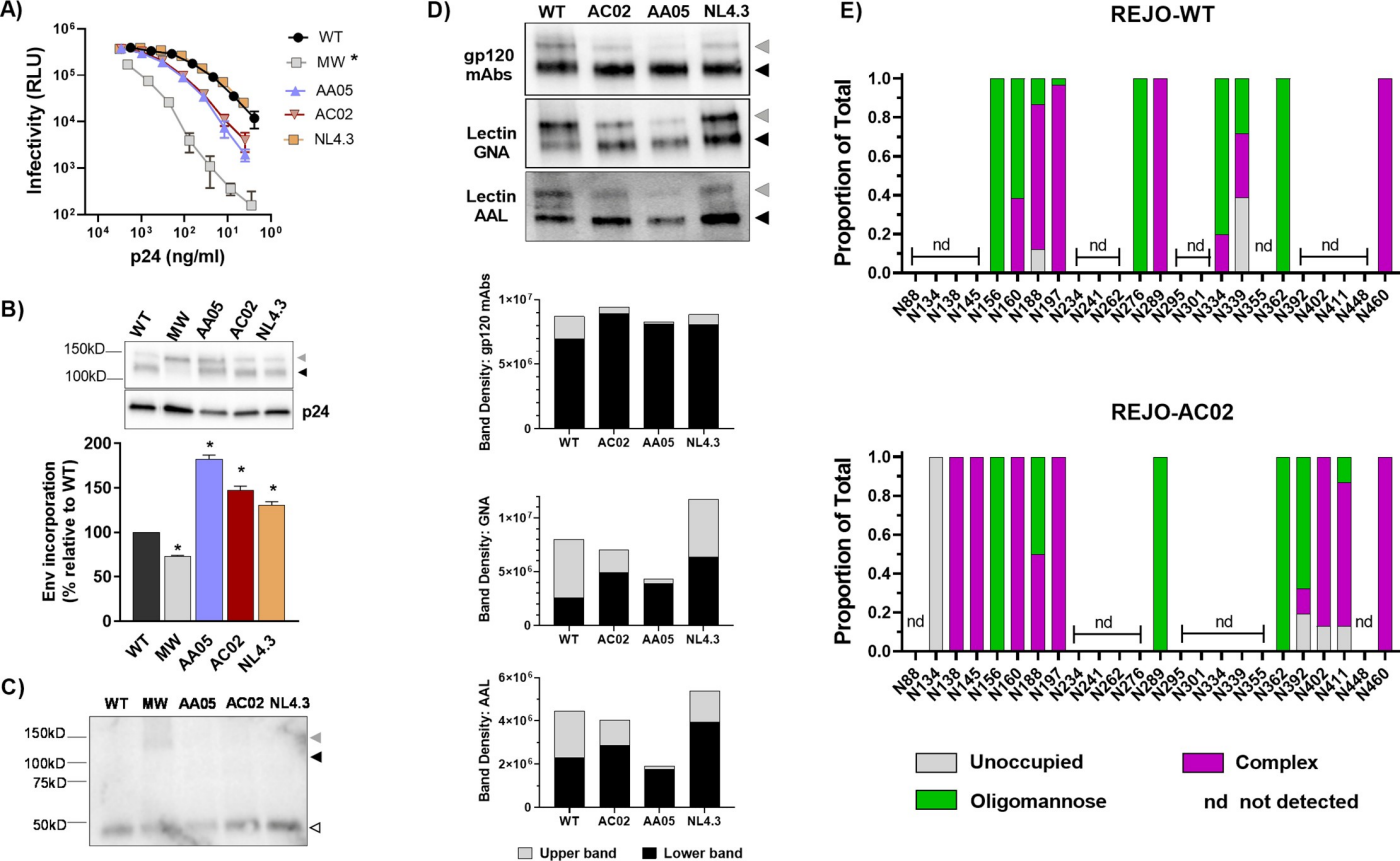
Effects of SP swap on REJO. (A) Infectivity of WT vs SP-swapped REJO derived from 293T cells in TZM.bl cells. *, p = 0.01 vs WT by Mann-Whitney test. (B) Measurement of Env incorporation into virions by Western blot. *, p values range from <0.0001 to 0.0016 vs WT by one-way ANOVA. (C) Western blot probed with gp41 MPER mAb 2F5. (D) Analysis of REJO Env glycosylation by lectin-probed Western blotting. (E) Relative amounts of unoccupied, oligomannose, and complex glycans at glycosites detected on the WT and AC02 SP-swapped REJO Envs as determined by LC-MS/MS. Gray, black and white triangles denote the positions of upper gp160, lower gp120, and gp41 Env bands, respectively.

To evaluate the effect of SP swapping on a different Env backbone, we tested a clade B tier 2 T/F IMC REJO and swapped its SP with SPs of MW965.26 (clade C, chronic, tier 1A), AA05 (clade B, acute), AC02 (clade B, acute), and NL4.3 (clade B, chronic, tier 1) (Fig 1). Except for REJO-MW965.26, infectivity of REJO with other SP swaps was not significantly altered, although REJO-AA05 and REJO-AC02 had slightly reduced infectivity (Fig 5A). MW965.26 SP lowered total Env incorporation, while SPs of AA05, AC02 and NL4.3 increased Env incorporation. Notably, the amount of cleaved functional Env (lower gp120 band) in REJO-MW965.26 was reduced by 90% (Fig 5B), corresponding to its low infectivity. The blot was also probed with gp41 mAb 2F5, which detected gp41 in all viruses and reacted weakly with the upper gp160 band of REJO-MW965.26 (Fig 5C).

We subsequently assessed the impact of SP switch on REJO Env glycosylation by lectin-probed Western blotting (Fig 5D). Comparable WT and SP-swapped total Env bands were detected by anti-gp120 mAbs. REJO-MW965.26 was excluded because of low Env content. In contrast, differences were observed with lectins (GNA and AAL) (Fig 5D). Compared with WT, the lower band of REJO-NL4.3 reacted more strongly with GNA (2.5-fold) and AAL (1.7-fold). Both GNA and AAL showed reduced binding to the upper band of REJO-AA05 and increased binding to the lower band of REJO-AC02. The LC-MS/MS analysis was able to detect 36% (10/28) and 46% (13/28) of glycosites present on virion-derived Envs of REJO-WT and REJO-AC02, respectively (Fig 5E). Comparison of seven glycosites detected in both Envs showed altered glycan occupancy at N160 and N188 on the V1V2 apex, as well as at N289 in the C2 region. While 62% of N160 was occupied by oligomannose in REJO-WT, it was 100% complex-type glycans on REJO-AC02. The oligomannose occupancy of N188 was 13% in REJO-WT and increased to 50% in REJO-AC02. N289 was completely switched from 100% oligomannose in WT to 100% complex in AC02. N156, N197, N362 and N460 glycans were not altered. Thus, as with CMU06, SP swapping of REJO also had an impact on Env glycan composition exemplified by significant changes in three out of seven detected glycans. LC-MS/MS did not detect many glycosites on REJO Envs, most likely due to the inadequate sensitivity of the technique and the relatively low abundance and purity of virion-derived Envs isolated by SDS-PAGE.

**Fig 6:**
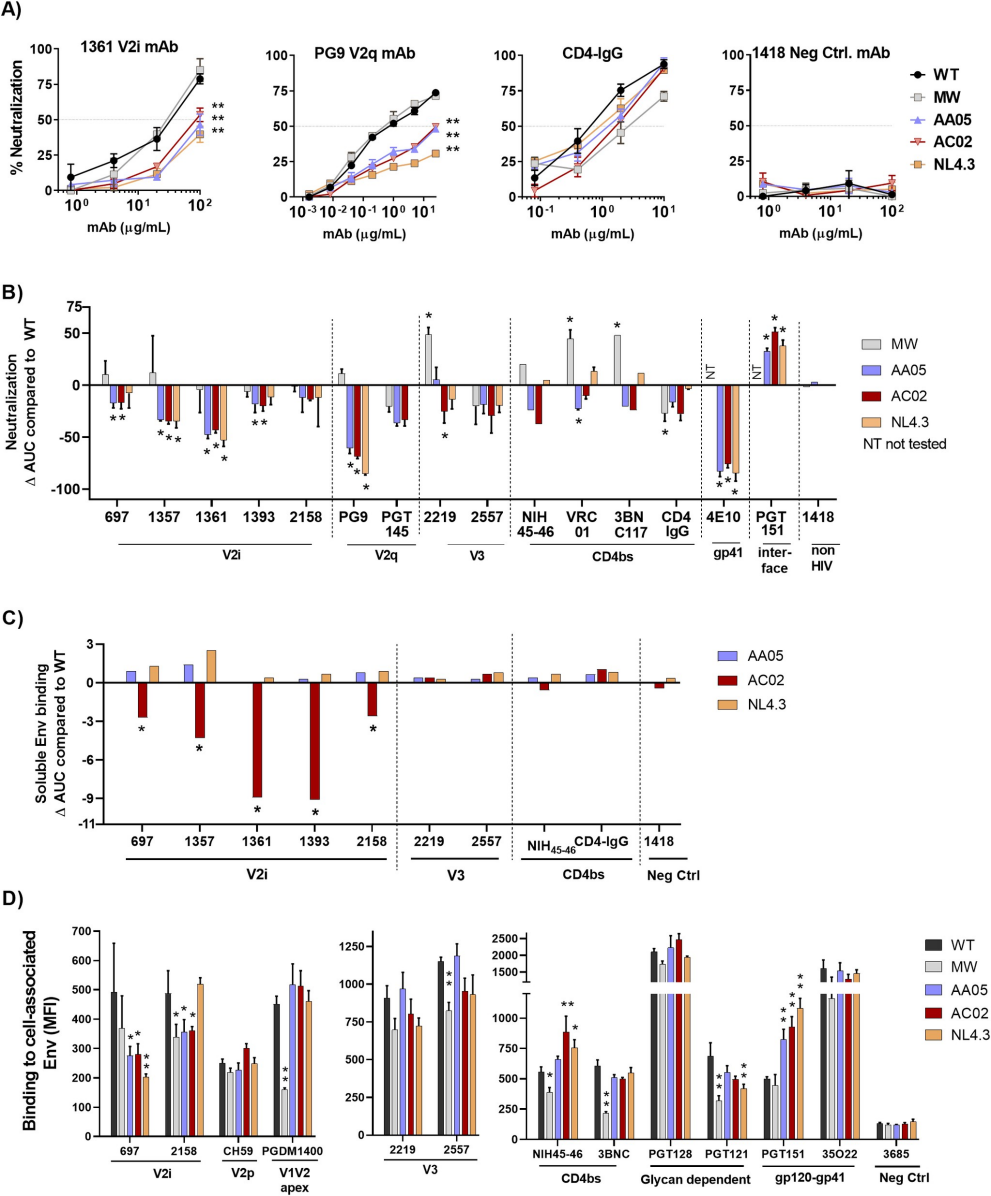
Effects of SP swap on REJO Env-antibody interaction. (A) Neutralization of WT and SP-swapped REJO treated with titrated amounts of anti Env mAbs. Neutralization was performed as in Fig 4A and 4B. Representative titration curves for V2i mAb 1361, V2q mAb PG9, CD4IgG and negative control mAb 1418 are shown. **, p< 0.001 vs WT by 2-way ANOVA. (B) Changes in neutralization AUCs of WT vs SP-swapped viruses by different mAbs. Statistical analysis was performed on neutralization curves (Fig 6A) by 2-way ANOVA (* p < 0.05) (C) ELISA binding of mAbs and CD4IgG to soluble Env from WT and SP-swapped viruses. Titration was done as in Fig 2C and AUC changes relative to the WT are shown. *, p< 0.05 vs WT by ANOVA of the titration curves. (D) Binding of mAbs to REJO WT vs SP-swapped Envs expressed on the surface of 293T cells. MAbs were tested at the following concentrations: 697, 2158 and 3685 at 100μg/ml; 2219 and 2557 at 50 μg/ml; PGT128, PGT121 and 35O22 at 25 μg/ml; CH59, 3BNC117 at 20 μg/ml; PGDM1400 and PGT151 at 10 μg/ml; NIH45-46 at 2.5 μg/ml. Averages and SEM of MFI from duplicates of two experiments are shown. *, p< 0.05; **, p< 0.01 vs WT by one-way ANOVA.

Similar to CMU06, virus sensitivity to neutralization was also altered for SP-swapped REJO vs WT. The effect was most pronounced for REJO with AC02, AA05 and NL4.3 SPs which became more resistant to most of the V2i mAbs tested (Fig 6A and 6B). These SP-swapped REJO viruses also became more resistant to V2q mAb PG9 and to lesser extent PGT145 (Fig 6A and 6B). In contrast, the sensitivity of REJO-MW965.26 to V2i and V2q mAbs was minimally altered. REJO-MW965.26, however, was more sensitive to V3 mAb 2219 and CD4bs mAbs VRC01 and 3BNC117. AA05, AC02 and NL4.3 SPs also rendered REJO more resistant to gp41 MPER mAb 4E10 and more sensitive to gp120-gp41 interface mAb PGT151. REJO-MW965.26 was not tested with 4E10 and PGT151.

To investigate the effect of SP swaps on REJO Env antigenicity, we assessed mAb binding to virus-derived Env (Fig 6C) and Env expressed on 293T cells (Fig 6D). Reduced binding of all V2i mAbs was seen with REJO-AC02. In contrast, binding of V3 mAbs, CD4bs mAb NIH45-46, and CD4IgG to REJO-AC02 was unaltered. REJO-MW965.26 was not examined due to low Env content. When we examined mAb binding to native Env on 293T cells, a different pattern was observed. Swapping MW965.26 SP onto REJO Env almost abrogated the binding of trimer-specific V2q mAb PGDM1400 and reduced the binding of V2i mAbs 697 and 2158, without affecting the binding of V2p mAb CH59. Compared with WT, REJO-MW965.26 also displayed reduced reactivity with CD4bs mAbs NIH45-46 and 3BNC117, and V3 glycan-specific PGT121. In contrast, REJO with AC02, AA05, and NL4.3 SPs had lower reactivity mainly with V2i mAbs (697 and/or 2158). PGT151 mAb, which binds specifically to the cleaved Env trimer, showed increased binding to these three SP swaps, while binding to REJO-MW965.26 was comparable with WT. Hence, like CMU06 SP swapping, REJO SP exchanges also modified Env interactions with mAbs.

### Strain-specific impact of SP swapping on SF162

**Fig 7:**
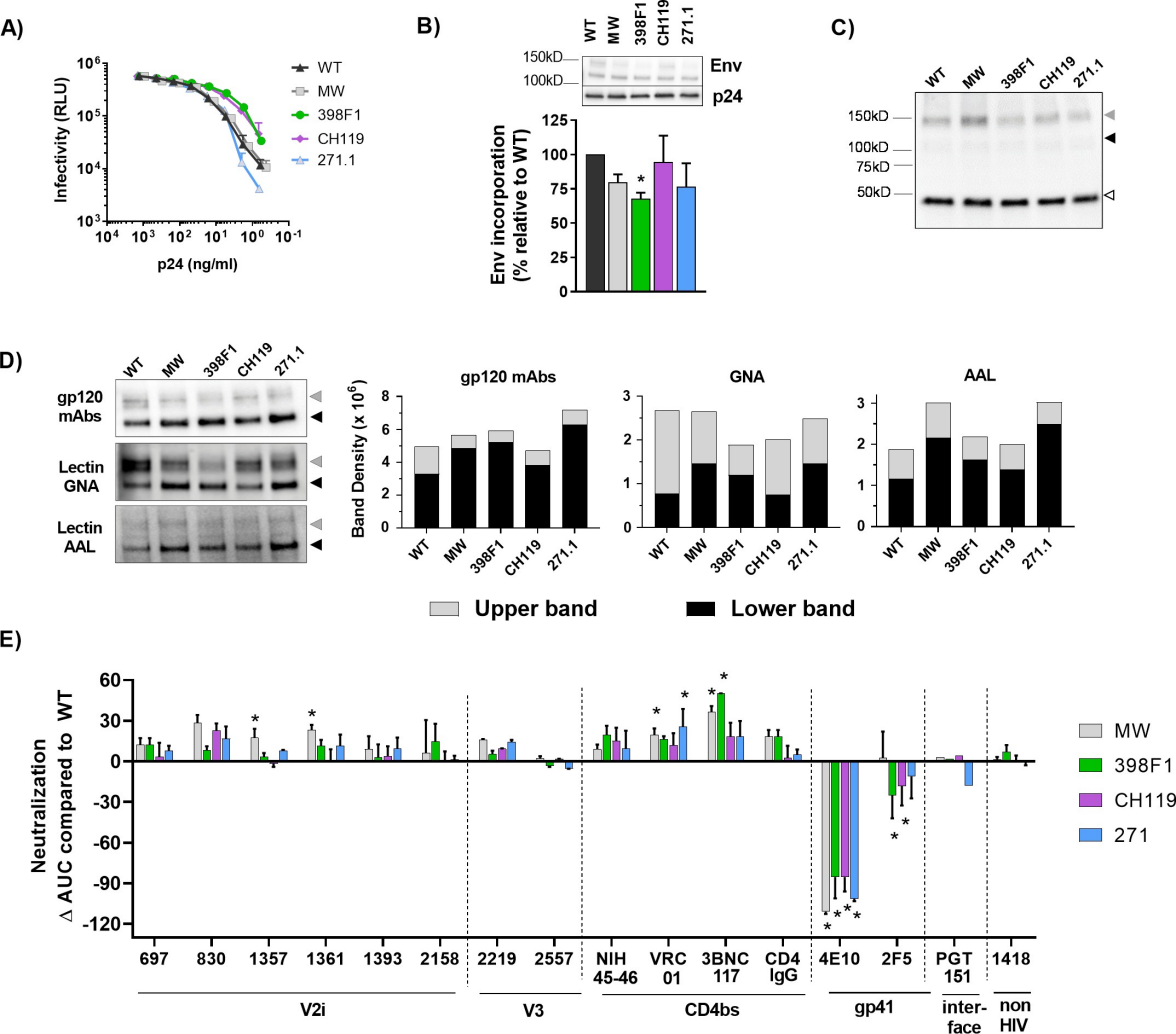
Effects of SP swap on SF162. (A) Infectivity of 293T-derived SF162 WT vs SP-swapped viruses in TZM.bl cells. (B) Measurement of Env incorporation by Western blot. *, p = 0.017 vs WT by unpaired t-test. (C) Western blot probed with gp41 MPER mAb 2F5. (D) Binding of Env from SF162 WT and SP-swapped viruses by gp120 mAbs and lectins (GNA and AAL) in Western blots. Densities of upper and lower Env bands are shown. (E) Changes in neutralization AUCs of SP-swapped vs WT viruses with different mAbs. Statistical analysis was performed on neutralization curves as in Fig 4A by 2-way ANOVA (* p < 0.05). Gray, black and white triangles mark the positions of upper gp160, lower gp120, and gp41 Env bands, respectively.

To assess the consequences of SP exchanges on another virus strain, we generated SF162 viruses with WT and SP-swapped Envs (Fig 1). SF162 is a tier-1A neutralization-sensitive, chronic, clade B isolate. All SP-swapped and WT SF162 viruses displayed comparable levels of infectivity (Fig 7A). Env incorporation was similar although a slight reduction was noted with 368F1 SP swap (Fig 7B). Western blots probed with gp41 mAb 2F5 also showed gp41 and gp160 bands without substantial reduction in intensity (Fig 7C). The GNA and AAL reactivity demonstrated moderate changes of oligomannose and fucose-bearing complex glycans on SP-swapped Envs vs WT (Fig 7D). However, in comparison with CMU06, the same SP exchanges did not make SF162 more resistant to V2i mAbs. The SP-swapped viruses were slightly more sensitive to neutralization by some V2i mAbs and CD4bs mAbs (Fig 7E). Compared to WT, MW965.26 SP especially made the virus more sensitive to V2i mAbs 1357 and 1361 and CD4bs mAbs VRC01 and 3BNC117. All four SP swaps made SF162 more resistant to MPER gp41 mAb 4E10, while SF162-398F1 and SF162-CH119 had increased resistant to mAb 2F5 (MPER gp41). Neutralization by V3 mAbs was not markedly changed.

**Fig 8:**
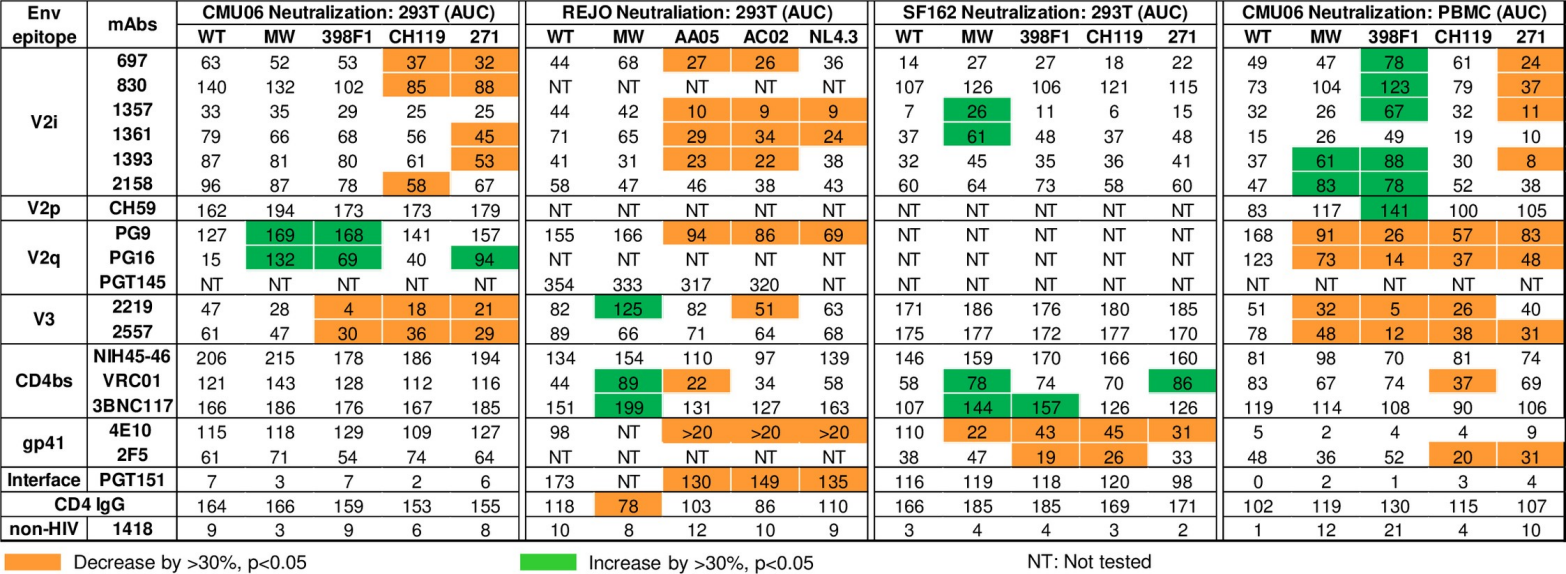
Neutralization AUC values of WT and SP-swapped viruses grown in 293T cells and PBMCs. AUCs values that decreased or increased by >30% and had p<0.05 relative to WT for the same MAbs or CD4IgG are shown in orange or green, respectively. NT: not tested.

Figs 8 and S2 summarize the differential effects of SP exchanges on neutralization phenotypes that depend on the Env backbones. These data illuminate the pronounced effects on neutralization by V1V2 mAbs: SP swaps reduced neutralization sensitivity of CMU06 and REJO, but not of SF162. SP swaps also affected the exposure of gp41 and CD4bs epitopes on SF162 and REJO Envs. The MW965.26 SP increased neutralization sensitivity to some V2i, V2q, V3, and CD4bs mAbs, but the effects varied depending on the Env backbones.

### Host-cell dependence of SP impact on glycosylation and neutralization

**Fig 9:**
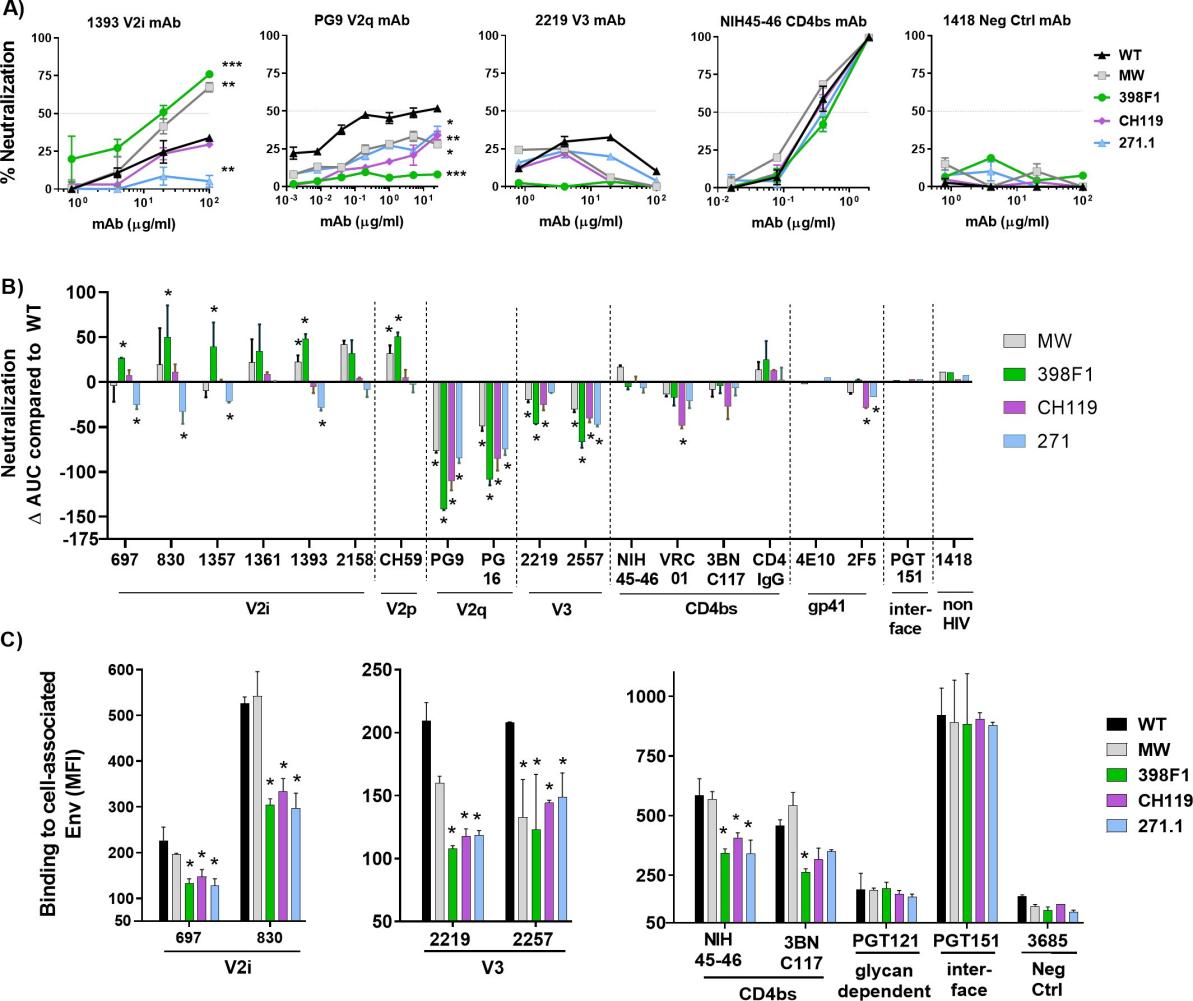
Effect of SP swap on neutralization of CMU06 produced in PBMCs and antigenicity of CMU06 Env expressed on human CD4^+^ T cells. (A) Virus neutralization was measured using TZM.bl cells. MAb 1418 served as a negative control. Neutralization curves of selected virus-mAb pairs are shown. *, p < 0.05; **, p < 0.01; ***, p < 0.001 vs WT by 2-way ANOVA (B) Changes in neutralization AUCs of SP-swapped vs WT viruses by different mAbs. Statistical analysis was performed on neutralization curves as in Fig 4A by 2-way ANOVA (* p < 0.05) (C) Binding of mAbs to Env expressed on CD4^+^ T cell surface. MAbs were tested at the following concentrations: 697, 830, 2219 and 2557 and 3685 at 100 μg/ml; PGT121 25 μg/ml; CH59 and PGT151 at 10 μg/ml; NIH45-46 at 2.5 μg/ml; 3BNC117 at 20μg/ml. MFI and SD from duplicates of one experiment are shown. *, p< 0.05 vs WT by ANOVA.

Because glycosylation is host cell-dependent [64,65], we investigated the effect of SP exchanges on CMU06 produced in primary CD4^+^ T cells. PBMC-derived SP-swapped CMU06 viruses displayed a radically distinct neutralization pattern from 293T-produced counterparts (Figs 8, 9A, 9B and S2). While 293T-derived SP-swapped CMU06 acquired increased resistance to V2i mAbs, PBMC-derived viruses with SPs from MW965.26 and 398F1 (both tier 1 isolates), but not CH119 and 271.1 (both tier 2), became more sensitive to neutralization by some or all V2i mAbs and V2p mAb. Remarkably, all PBMC-derived SP-swapped viruses became more resistant to V2q mAbs PG9 and PG16, although neutralization by most CD4bs mAbs and CD4IgG was largely unaltered (Fig 9B). Only swapping with CH119 SP reduced sensitivity to CD4bs mAb VRC01 and MPER gp41 mAb 2F5, without affecting neutralization by V2i and V2p mAbs. V3 crown mAbs had neutralization <50% against CMU06-WT, and the SP-swapped viruses were even more resistant than WT (Fig 9B). PBMC-derived CMU06 viruses were not neutralized by the gp120-gp41 interface mAb PGT151 (IC50 >20μg/ml) and 4E10 (IC50 >10μg/ml).

SP swapping also altered the antigenicity of Env expressed on primary CD4^+^ T cells. The changes were more drastic on SP-swapped CMU06 Envs expressed on CD4^+^ T cells (Fig 9C) vs 293T cells (Fig 4D). V2i mAbs bound CMU06-398F1, CMU06-CH119, and CMU06-271.1 less than CMU06-WT, but binding to CMU06-MW965.26 was unaltered. These SP-swapped viruses also showed reduced binding with V3 mAbs (2219 and 2557), and CD4bs mAbs (NIH45-46 and 3BNC117), although binding of PGT151 and PGT121 was comparable. Altogether, the data show that SP swapping altered the antigenicity of native Env present on virions and cells, albeit in an Env strain- and host cell-dependent manner. Notwithstanding these variabilities, SP swapping clearly affects different Env epitopes.

**Fig 10:**
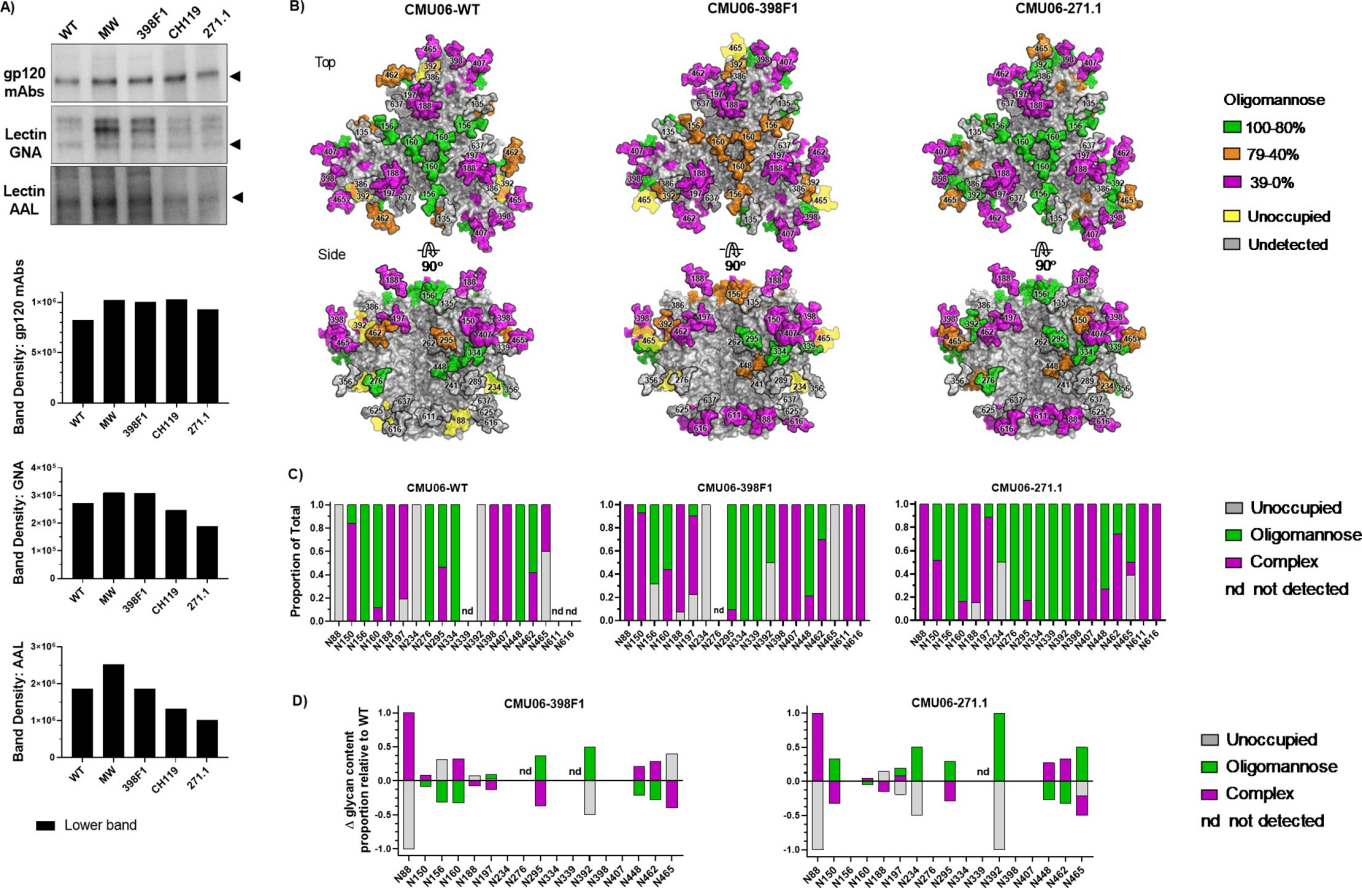
Changes in glycosylation on Env of CMU06 WT and SP-swapped viruses produced in PBMC. (A) Binding of Env by mAbs and lectins (GNA and AAL) measured by Western blots. Black triangles denote the lower gp120 Env bands detected on the blots. (B) Models of glycosylated Env trimers from PBMC-derived CMU06 WT and SP-swapped viruses. Models and glycans were generated as in Fig 3A. (C) Proportions of unoccupied, oligomannose, and complex glycans at each glycosite. Glycosites detected in at least one of three Envs are listed. nd: not detected in 1 or 2 Envs. (D) Changes in glycan content at each glycosite for CMU06-398F1 and CMU06-271.1 vs CMU06-WT calculated from data in Fig 10C.

We further determined glycosylation changes on SP-swapped Envs from PBMC-produced CMU06 using lectin-probed Western blot. Unlike 293T-derived viruses (Fig 2D), we detected predominantly the lower gp120 bands with anti-gp120 mAbs (Fig 10A), suggesting host cell-specific differences in gp120/gp41 processing. Therefore, we analyzed GNA and AAL binding to the lower bands only. Moderate differences were observed in GNA and AAL binding to SP-swapped vs WT CMU06 (Fig 10A). However, the binding patterns were distinct from those seen with 293T-derived CMU06 (Fig 2D).

We also conducted glycosite-specific analyses of Envs from PBMC-grown viruses by LC-MS/MS (Fig 10B–10D). Of 28 N-glycosites in CMU06 gp160, we detected 16, 18 and 19 in CMU06-WT, CMU06-398F1 and CMU06-271.1, respectively. When the 15 glycosites detected in all three Envs were compared, the percentages of unoccupied glycans was higher in CMU06-WT (25%) and CMU06-398F1 (21%) compared to CMU06-271.1 (7%) (Figs 10B and S3). The oligomannose glycans were higher in CMU06-271.1 (48%) compared to CMU06-WT (34%) and CMU06-398F1 (33%). Likewise, total abundance of complex glycans was also altered in CMU06-398F1 (47%) and CMU06-271.1 (45%) vs CMU06-WT (40%).

We noted that changes in overall oligomannose glycans in PBMC- and 293T-derived viruses correlated with altered sensitivity to V2i mAbs. The total oligomannose content increased by 4.4% and 7.9% on 293T-derived CMU06 viruses with 398F1 and CMU06-271.1 SP respectively and by 10.1% on PBMC-derived CMU06-271.1 over that of CMU06-WT (S3 Fig). Correspondingly, these SP-swapped viruses showed a trend of or a significant increase in V2i mAb resistance vs WT: the cumulative neutralization AUC values decreased by 87 and 188 for 293T-derived CMU06-398F1 and CMU06-271.1, respectively, and by 125 for PBMC-derived CMU06-271.1. In contrast, the oligomannose content of PBMC-derived CMU06-398F1 was reduced by 5.7% and the virus was more sensitive to V2i mAbs (cumulative AUC of 483 vs 253 for WT). Changes in complex glycans, on the other hand, correlated with altered sensitivity to V2q mAbs. A decrease in complex glycan content was observed in 293T-derived CMU06-398F1 and CMU06-271.1 by 3.1% and 10.6% respectively, and these viruses were more sensitive to V2q mAbs (cumulative AUC of 237 and 251 vs 143 for WT). Conversely, when the viruses were grown in PBMCs, the total complex glycans increased by 12.1% and 8.1%, and the viruses were more resistant to V2q mAbs (cumulative AUC of 40 and 131 vs 290 for WT).

Analyses of individual glycosites on PBMC- and 293T-derived viruses revealed three patterns. First, changes were consistently observed at N160 on the V1V2 apex in SP-swapped CMU06 produced in both cell types (Figs 3, 10 and S3). N160 had a decreased oligomannose content upon 398F1 SP swapping, regardless of the producer cells. Second, we observed glycans that were affected by both host cells and SPs; these comprised most N-glycans detected: N88, N150, N156, N188, N197, N295, N392, N407, N448, N462, N465, N611 and N616. Glycan content at these glycosites differed for PBMC- vs 293T-produced viruses and among the three SP-swapped viruses (S3 Fig). Notably, N156 on the V1V2 apex displayed only oligomannose glycans in PBMC-derived CMU06-WT, but had a mix of oligomannose and complex glycans in 293T-derived CMU06-WT. On PBMC-derived CMU06-271.1, N156 also was completely occupied with oligomannose, whereas on 293T-derived counterpart it was mostly unoccupied. N156 had reduced oligomannose contents on CMU06-398F1 vs CMU06-WT, when produced in PBMC or 293T cells. Another example is illustrated by N88 glycan at the trimer base, which was completely unoccupied in PBMC-derived CMU06-WT, but 82% unoccupied in 293T-derived WT. In contrast, N88 was populated exclusively by complex glycans in CMU06-398F1 and CMU06-271.1 from PBMC, although it was 50% and 80% unoccupied in 293T-derived counterparts. Third, glycan changes were found that were dependent on host cells only and independent of SPs. For example, 100% complex glycans occupied N398 on all three PBMC-derived viruses, these were reduced to 22–36% in 293T-derived viruses.

Few glycosites were not identified in all samples, allowing only partial comparison. For example, N334 had only oligomannose glycans on all PBMC-derived viruses and 293T-derived CMU06-398F1. N611 on gp41 was detected in two PBMC-derived and three 293T-derived viruses and displayed exclusively or mostly complex glycans. N616 had complex glycans on four of five PBMC- and 293T-derived viruses but a mix of complex and oligomannose glycans on CMU06-271.1 from 293T cells.

Collectively, these data revealed the impact of SP changes on glycan compositions at specific glycosites and on Env interactions with mAbs. The results also demonstrated distinct SP-induced glycan alterations on viruses from PBMCs vs 293T cells. Because we compared viruses with identical Env sequences, the observed differences reflect how glycosylation proceeds at each glycosite in the context of different SPs and host cells.

## Discussion

HIV-1 Env glycosylation is influenced by viral and cellular factors. This study demonstrates the effect of Env SP, a highly variable albeit understudied viral element, on glycosylation and consequently on Env properties and virus phenotypes. Introducing SPs from different virus strains to the same Env backbone altered proportions of oligomannose, complex, and unoccupied glycans on multiple glycosites. Interestingly, SP exchanges altered the oligomannose content of N156 and N160 glycosites on V1V2 at the Env spike apex essential for the V2q-class bNAbs. These effects were present in all virus-derived Envs analyzed by LC-MS/MS, regardless of the producer cells (PBMC and 293Tcells). Pronounced effects of SP swapping were also observed on several glycans within the gp120-gp41 interface at the trimer base. Correspondingly, SP exchanges affected Env recognition and virus neutralization by mAbs, including those targeting V1V2, V3, and gp41 MPER epitopes. Thus, although SP is not a part of the mature Env, it is a viral determinant that affects Env glycosylation and antibody recognition.

Among the three classes of V1V2 mAbs, V2i and V2q mAbs were prominently affected by SP swaps, as evidenced by altered sensitivity of many SP-swapped tier-2 CMU06 and REJO viruses to neutralization by these mAbs. The effects nonetheless varied depending on SPs and host cells producing the virus. Reasons for altered sensitivity to V2i mAb-mediated neutralization are not fully understood. The V2i epitopes are often occluded on native pre-fusion trimers of tier-2 viruses [19] and become exposed to mAbs only after prolonged virus-antibody incubation [11]. The structural dynamics influencing V2i epitope exposure is governed partly by Env glycan composition. Our previous studies showed that enrichment of high-mannose glycans increased virus resistance to V2i mAbs, even when the mAb-virus incubation was extended to >18 hours [27]. Here, we observed that SP swaps increased or decreased total oligomannose content to associate with increased or decreased resistance to V2i mAbs, although the presence of distinct glycan profiles also incurs variability in Env conformation and recognition by mAbs specific for other epitopes. These data suggest that modulating oligomannose glycans is one potential strategy that HIV-1 can employ to resist neutralizing activities of V2i and other Abs, and such alteration can be triggered by changes in Env SP. Consistent with this, a single substitution in the N-terminal SP region was found sufficient to affect Env glycan composition and increase virus resistance to neutralization by V2i mAbs, V2q mAbs, mannose-binding mAb 2G12, or CD4-IgG [27].

Unlike V2i mAbs, V2q mAbs PG9 and PG16 directly target oligosaccharide moieties as part of their epitopes [66]. Hybrid glycans bearing high mannose and biantennary motifs with two terminal sialic acids at N156 and N160 on the V1V2 apex are important for PG9 binding, whereas PG16 recognizes α-2,6-sialylated hybrid and complex glycans on these sites [17,67]. SP swap affected virus sensitivity to V2q mAbs. An SP swap-induced shift in glycan content may supplant the hybrid-type glycan structures with terminal moieties preferred by these V2q mAbs [17,67], although this could not be resolved in this study. Importantly, our LC-MS/MS data offered evidence for the first time that the modulation of virus sensitivity to these V2q mAbs correlated with changes in the complex glycan content on the viruses. The data indicate that an increased amount of complex glycans is unfavorable for V2q mAbs. Nonetheless, glycans proximal to N156 and N160, such as N136 and N149 (undetected here) can also influence the binding and neutralization by V2q mAbs [17,68]. While more complete data are needed to define Env glycomes compatible with V2q mAbs, the data clearly shows that the glycan compositions critical for V2q mAbs are influenced by the SP sequences.

Env binding and virus neutralization by CD4IgG were unperturbed, indicative of the proper folding of all tested SP-swapped Envs. The SP-swapped Envs also maintained the features characteristic of the native trimeric Env, such as the presence of TAMP, which is absent on uncleaved or incorrectly assembled trimers [69–71], and the exclusive oligomannose content at N276 when this glycosite was detected (PBMC-produced CMU06 with WT and 271.1 SP). This contrasted to the N276 occupation by complex glycans on BG505 pseudotrimers and gp120 monomers [69]. Further, we observed no drastic reduction of PGT151 mAb binding to SP-swapped vs WT Envs. Three SP swaps even increased PGT151 binding to REJO Env, and correspondingly these viruses were also more sensitive to neutralization by PGT151. PGT151, which targets glycan-bearing epitopes in the gp120-gp41 interface like mAb 35O22, binds only to properly folded, cleaved trimers [61,72]. Similarly, Env binding and virus neutralization by trimer-dependent V2q mAbs PGT145 and PGDM1400 were largely undisturbed by SP exchanges, except for REJO-MW965.26. We also observed only minimal sporadic effects on neutralization by V3 crown mAbs, demonstrating that SP swapping did not shift Env trimers toward more open conformations [73–75]. By contrast, prominent changes were observed in neutralization of REJO and SF162, but not CMU06 viruses, by MPER mAb 4E10, although the reasons remain unknown and warrant further studies. Altogether, these data indicate that SP swapping subtly modifies the proportion of Env oligosaccharide content and the antigenicity of Env without triggering massive structural transformations.

Except for REJO-MW965.26, SP exchanges did not seriously affect virus infectivity. The decreased infectivity of REJO-MW965.26 can be explained by the diminished expression of functional cleaved Env gp120 and its incorporation into virions. However, this effect was not seen when MW965.26 SP was introduced to CMU06 or SF162. The reasons for REJO-MW965.26 incompatibility are still unclear. A reduction of cleaved gp120 was previously noted when glutamine (Q) was introduced to position 12 of REJO SP (H12Q), but not to that of JRFL SP (Y12Q) [27]. Glutamine occupies position 12 of MW965.26 SP and not the other SPs tested with REJO, but MW965.26 SP also differs from REJO SP at 9 other positions, requiring systematic evaluations for the contribution of glutamine at position 12 vs other MW965.26 SP residues. We also observed that the MW965.26 SP imparted a more sensitive phenotype to REJO in the context of V3 and CD4bs mAbs, but this effect was not observed on CMU06. Moreover, two other tier 1 SPs (398F1 and NL4.3) did not confer REJO or CMU06 sensitivity to V3 and CD4bs mAbs. Nonetheless, this study was limited to testing only few tier 1 SPs for each Env backbone.

Glycosylation begins in the ER with the addition of Glc3Man9GlcNAc2 onto asparagine in the NX(T/S) (X≠Proline) sequon on a nascent protein. As the protein crosses the ER and the Golgi apparatus, the high-mannose structure is trimmed and subsequently elaborated with hybrid- and complex-type glycans. In view of the observed effect of SP swapping, we suggest that the extent to which the glycan on a particular glycosite is processed is decided while Env is an ER resident with its SP still tethered. SP may subtly affect the compactness of Env folding, which consequently imposes or releases structural constrains to enzymes that generate hybrid or complex glycans in the Golgi. Nonetheless, the steps at which the SP sequence dictates the glycan content of Env are unknown. The delayed post-translational SP cleavage is conserved across HIV-1 subtypes ensuring low Env expression on virions [31,38,75]. In addition to the positively charged n-region, residues in the hydrophobic h-region and downstream of the SP cleavage site are important for this property [31,38]. The h-region forms an α-helix across the ER membrane and covers the cleavage site to delay SP cleavage [31,37]. Since the cleavage sites in SP-swapped Envs and WT counterparts are identical, perhaps the h-region variations affect the cleavage site accessibility for signal peptidase, altering the efficiency of SP cleavage of Env with heterologous SPs, similar to that seen with the h-region-mediated regulation of SP cleavage for preprolactin [76,77].

Prediction based on Model SignalP 3.0 [78] indicates that the cleavage probabilities were slightly increased for CMU06 with 398F1, CH119, and 271.1 SPs vs WT (S4A and S4B Fig), with 271.1 SP having the highest probability. Topology and structure predictions using PSIRED [79,80] show differences in the cytoplasmic and ER regions and in the α-helical structure around the cleavage site (S4C and S4D Fig). However, experimental data are needed to verify if cleavage efficiency is indeed changed to affect the rate of Env synthesis. Past studies have shown that replacing SPs of influenza hemagglutinin and HIV-1 Env by tPA/22P SP increased SP cleavage probability to 0.925, leading to higher secretion of these proteins [81]. Similarly, M26P mutation in HIV-1 HXB2 SP was found to increase the SP cleavage probability from 0.628 to 0.928 and change the SP cleavage from post-translational to co-translational [38]. Prolines are notably absent near SP cleavage sites in 99.9% of HIV-1 gp160 [38].

In additional to SP cleavage, SP interaction with the signal-recognition particle (SRP) may also play a role in determining Env phenotypes. The h- and n-regions, which vary greatly among SPs in length and sequence, are deemed important for SRP interaction [82–84]. Thus, it is possible that different SPs may bind SRP with different affinities impacting the chaperone-mediated Sec61 channel gating and the interaction with chaperones involved in protein folding and assembly [76,85,86]. A single substitution of a helix-breaking glycine residue by a helix-promoting leucine residue in the hydrophobic core of prokaryotic SPs can promote SRP binding [87,88] and regulate the pathway of protein translocation [89]. The SPs studied here differ in the number of basic, hydrophobic, and leucine residues (Fig 1); the significance of each element needs to be better understood, considering that SP exchange is a common strategy to promote secretion of recombinant Env vaccines and SP selection is critical to generate glycomes faithfully representing those of native Envs.

In summary, this study demonstrates an important role of HIV-1 Env SP in influencing Env glycan content. By introducing SP from a particular HIV-1 strain, we can modulate the relative proportion of unoccupied, high-mannose, and complex glycans on specific glycosites, including those on V1V2 at the apex of Env spike and on the gp120-gp41 interface near the base. SP swaps also can alter Env recognition and virus neutralization by mAbs against V1V2, gp41, and other epitopes. Data from this study have significant implications for vaccine development: SP is a critical component that must be rationally selected and incorporated into the design of Env-based HIV-1 vaccine.

## Materials and methods

### Plasmids

Infectious molecular clones (IMC) were generated by cloning the CMU06 and SF162 Envs into pNL4.3 backbone to construct pNL-CMU06 and pNL-SF162, respectively. To generate SP-swapped CMU06 IMCs, restriction site NheI was introduced at positions 89–90 (CGGAGT to GCTAGC) without amino acid alterations (A29, S30). DNA fragments with swapped SPs were synthesized (Genscript) and digested using EcoRI (in pNL4.3 backbone) and NheI and ligated to pNL-SF162 and pNL-CMU06 digested with the same enzymes. The pREJO.c/2864 IMC (REJO) was used to make REJO SP-swapped plasmids by a multi-step overlapping PCR mutagenesis strategy using Pfx50™ DNA Polymerase PCR System [27]. Briefly, in the first PCR step, mutated fragments were individually generated in two separate reactions. The primer pairs AvrIIF/MWR and MWF/BstEIIR were used to generate pREJO-MW (STAR Methods). The PCR fragments were agarose gel-purified, combined, and added to a second-stage PCR with the flanking primers AvrIIF and BstEIIR. Products of the second-stage PCR were digested by AvrII and BstEII restriction enzymes and inserted into the AvrII- and BstEII-digested fragment of pREJO.c/2864 to yield REJO-MW. The other REJO plasmids were constructed similarly using the primers listed in STAR Methods. All the plasmids were sequenced to confirm the presence of the desired sequence changes without any other mutations.

### Cell lines

HEK293T/17 cells (293T) were used to produce infectious HIV-1 viruses. TZM.bl cell line was used to assay virus infectivity and virus neutralization. TZM.bl cell line is derived from HeLa cells and genetically modified to express high levels of CD4, CCR5 and CXCR4 and contain reporter cassettes of luciferase and β-galactosidase that are each expressed from an HIV-1 LTR. The 293T and TZM.bl cell lines were routinely subcultured every 3 to 4 days by trypsinization and were maintained in Dulbecco’s modified Eagle’s medium (DMEM) supplemented with 10% heat-inactivated fetal bovine serum (FBS), HEPES pH 7.4 (10 mM), L-glutamine (2 mM), penicillin (100 U/ml), and streptomycin (100 μg/ml) at 37°C in a humidified atmosphere with 5% CO2.

### Viruses

Infectious viruses were generated by transfecting 293T cells with wild type (WT) or SP-swapped pNL-CMU06, pREJO and pNL-SF162 plasmids using jetPEI transfection reagent [27]. Supernatants were harvested after 48 hours and clarified by centrifugation and 0.45μm filtration. Single-use aliquots were stored at −80°C until use. Virus infectivity was assessed on TZM.bl cells as described [27,90]. Briefly, serial two-fold dilutions of virus stock in 10% DMEM were incubated with TZM.bl cells (in duplicates for each dilution) in half-area 96-well plate in presence of DEAE-Dextran (12.5 μg/ml) for 48 hours at 37°C. Virus infectivity was measured by β-galactosidase activity. The dilution of a virus stock that yielded RLUs between 150,000 and 200,000 was used in neutralization assays, as detailed below. HIV-1 p24 ELISA assay was used to quantify the p24 content in each virus stock using the manufacturer's protocols.

To generate PBMC-derived viral stocks, PBMC were isolated by lymphoprep gradient centrifugation from leukopaks of HIV-1 seronegative donors. Prior to HIV-1 infection, PBMC were activated by incubation in RPMI-intereukin-2 (IL-2) growth medium containing 10 μg of phytohemagglutinin (PHA) (PHA-P) per ml. The RPMI-IL-2 growth medium was RPMI 1640 medium supplemented with 10% heat-inactivated fetal calf serum, L-glutamine (2 mM), penicillin (100 U/ml) and streptomycin (100 μg/ml) and 20 U of recombinant IL-2 per ml. After overnight incubation with PHA, cells were washed and cultured with IL-2 for additional 2 to 3 days. All cultures were maintained in 5% CO2 incubators at 37°C. PBMCs were exposed to virus (50ng p24 of 293T-derived stock) overnight and then washed to remove the viral inoculum. Virus-containing supernatant was harvested on day 7 and day 10. Virus infectivity and p24 contents were measured as above.

### Human monoclonal antibodies

V2i, V3, and control mAbs were produced in our laboratory as described [23,91–97]. Other mAbs used in this study were obtained through the NIH AIDS Reagent Program, Division of AIDS, NIAID, NIH. The following antibody reagents were obtained through the NIH AIDS Reagent Program, Division of AIDS, NIAID, NIH: The following reagent was obtained through the NIH AIDS Reagent Program, Division of AIDS, NIAID, NIH: Anti-HIV-1 gp120 monoclonal PG9, PG16 PGT145, PGT121, PGT128, from IAVI [26]; anti-HIV-1 gp120 monoclonal CH59 from Drs. Barton F. Haynes and Hua-Xin Liao [21]; anti-HIV-1 gp120 monoclonal VRC01 from Dr. John Mascola [98]; anti-HIV-1 gp120 Monoclonal b12 from Dr. Dennis Burton and Carlos Barbas [99]; anti-HIV-1 gp120 monoclonal 3BNC117 from Dr. Michel C. Nussenzweig [100]; anti-HIV-1 gp120 monoclonal 17b from Dr. James E. Robinson [101]; anti-HIV-1 gp120 monoclonal 2G12 from Polymun Scientific [102]; anti-HIV-1 gp41/gp120 monoclonal 35O22, from Drs. Jinghe Huang and Mark Connors [102]; anti-HIV-1 gp41/gp120 monoclonal PGT151 from Dennis Burton; anti-HIV-1 gp41 monoclonal 2F5 and 4E10 from Polymun Scientific [103, Stiegler, 2001 #1724]}. The V2i, V3, gp41 mAbs were produced in our laboratory as described [23,91–97]. An CD4IgG fusion protein was also included, along with an irrelevant anti-parvovirus mAb 1418 and anti-anthrax mAbs 3685 used as negative controls.

### Virus neutralization assay

Neutralization activity of HIV-1-specific antibodies was measured as a reduction in β-galactosidase reporter gene expression of TZM-bl cells after infection of TZM-bl cells with WT and SP-swapped viruses. Neutralization was performed using either the standard 1 hour assay [11,27,103] or the prolonged 24 hour assay [11,103]. Serially diluted antibodies and viruses (150,000–200,000 RLUs) were pre-incubated for 1 hour or 24 hours prior to adding the target TZM.bl cells. After a 48-hour incubation at 37°C and 5% CO2 incubator, the β-galactosidase activity was measured. Each condition was tested in duplicate or triplicate, and experiments were repeated 2–4 times. Percent neutralization was determined based on virus control (TZM.bl cells with virus alone) and cell control (TZM.bl cells only) under the specific assay condition. Neutralization assays were performed after a 24-hour mAbs-virus incubation for all mAbs except PG9, PGT145, NIH45-46, VRC01, 3BNC117, and CD4IgG which were pre-incubated with viruses for 1 hour.

### Western blot analyses with antibodies and lectin probes

Western blot analyses were performed to quantify the ratios of Env to p24 proteins incorporated into the WT and SP-swapped viruses and to evaluate Env reactivity with different lectins as in [27]. The virus particles sucrose-pelleted from supernatants were lysed in SDS-PAGE loading buffer, resolved on 4–20% tris-glycine gels, and blotted onto nitrocellulose membranes, blocked overnight with 5% skim milk powder in phosphate buffered saline (PBS) followed by probing with antibodies or lectins. Anti-human anti-gp120 mAbs (a cocktail of anti-V3: 391, 694, 2219, 2558; anti-C2: 846, 1006; anti-C5: 450, 670, 722, 1μg/ml each) and anti-gp41 mAb (2F5) were used to detect Env. The mAb 91-5D (1μg/ml) was used to detect Gag p24. Biotinylated GNA, and biotinylated AAL were each used at 2μg/ml. Lectin binding was detected with HRP-neutravidin (1:1500 for 1 hr RT). All dilutions were made in Superblock T20 Buffer. Membranes were developed with Clarity Western ECL substrate and scanned by ChemiDoc Imaging Systems (Bio-Rad Laboratories). Purified recombinant gp120 and p24 proteins were also loaded at a known concentration as controls (data not shown). Band intensities were quantified using the Image Lab Software Version 5.0.

### Soluble Env binding assay

The relative binding of mAbs to solubilized Env from WT and SP-swapped viruses was measured by a sandwich ELISA. Half-area high-binding ELISA plates were coated with sheep anti-C terminal gp120 Abs (1μg/ml in PBS), blocked with 2% bovine serum albumin (BSA) in PBS, and incubated with 1% Triton X100-virus lysates containing 20 ng/ml Env (quantitated by Western blots). Serially diluted mAbs (0.01–10μg/ml) were then added for 2 hours, and the bound mAbs were detected with alkaline phosphatase-conjugated goat anti-human IgG and p-nitrophenyl phosphate substrate. The optical density (OD) was read at 405nm using BioTek PowerWave HT Microplate Spectrophotometer.

### Cell-associated Env binding assay

Assay to detect antibody binding to cell surface-expressed Env was performed as described [104] with minor modifications. A total of 4 × 10^6^ 293T cells were seeded in 15 ml of culture medium in a 100-mm tissue culture dish and incubated at 37°C. Twenty four hours later, cells in each dish were transfected with 20 μg gp160 expression plasmid (WT or SP swaps) using JetPEI (DNA:JetPEI ratio of 1:3) following manufacturer’s instructions. The transfected cells were incubated for 24 hours at 37°C, washed with PBS, detached with trypsin-free cell-dissociation buffer, and resuspended in PBS containing 2% BSA. Cells were stained with Live/dead Aqua stain and distributed into 96-well round-bottom tissue culture plates (5x10^4^/well) for individual staining reactions. Cells were incubated with mAbs at concentrations detailed in figure legends. For detection of mAb binding, biotinylated goat anti-Human IgG Fc (1:1000) followed by streptavidin phycoerythrin (PE) (1:500) was used. The cells were washed 3X with PBS-B (PBS plus 1%BSA) after each step and all incubation steps were performed on ice for 30 min. Cells were analyzed with a BD Fortessa flow cytometer, and 30,000 events were collected in the PE+ gate. Analysis was carried out using FCS-Express software as follows: 293T cells were selected from a plot of forward-area vs. side scatter-area (FSC-A/SSC-A) from which doublets were excluded in a forward scatter height vs forward scatter area plot (FSC-H/FSC-A). Live cells were selected by Aqua-negative gating, and geometric mean fluorescent intensity (MFI) of PE+ cells, representing anti-Env-stained cells, were quantified. Background MFI, as determined from cells stained without primary antibodies was subtracted from all Env-mAb pairs.

For testing mAb binding to Env expressed on primary CD4+ T cells, the experiments were conducted as above with the following modifications: CD4+ T cells were isolated from PBMCs (isolated from Leukopaks) using an EasySep Human CD4+ T cell Enrichment Kit. PHA-activated CD4+ T cells were infected with WT or SP-swapped viruses (500 ng p24/million cells) by spinoculation at 1200×g for 2 hours. The virus inoculum was replaced with fresh medium (RPMI 1640 media supplemented with 10% FBS with 20 U/mL IL-2) and the cells were further incubated for 7 days at 37°C. Cells were harvested for mAb staining as above.

### Mass spectrometry

Analysis for site-specific glycosylation was performed as in [105]. Briefly, 293T- and PBMC-derived infectious virus stocks were concentrated by sucrose cushion centrifugation. Concentrated (250X) virus preparations were loaded on SDS-PAGE gel (7.5%) and the separated Env bands were excised to use directly for mass spectrometry (MS). The SDS-PAGE gel bands were washed with 100% acetonitrile and water three times. A proteomics-based strategy was used to assess the degree of glycan processing and the degree of site-occupancy of each glycosite [105]. The proteins in the gel bands were denatured and alkylated by 10 mM dithiothreitol (DTT) and 55 mM iodoacetamide in 25 mM ammonium bicarbonate, respectively. The resulting proteins were digested with the combination of trypsin and chymotrypsin at an enzyme/substrate ratio of 1:15 (w/w) and 1:10 (w/w) respectively in 25 mM ammonium bicarbonate. Sequential treatment with two endoglycosidases was then performed to introduce novel mass signatures for peptides that contain glycans of high-mannose types and complex-type glycans [105]. First, the Env peptides were digested with Endo H to cleave high-mannose (and hybrid) glycans between the innermost GlcNAc residues, leaving a GlcNAc attached to Asn (N+203). The subsequent PNGase F treatment removed the remaining complex-type glycans, and in the process converted Asn to Asp, resulting in a +0.984 Da mass shift (N+1) [106,108]. For peptides with unoccupied glycosites, these treatments produce no mass shift (N+0). Using this strategy, liquid chromatography–mass spectrometry (LC-MS/MS) data were acquired for each sample. Peptides were identified using SEQUEST. The abundance of each peptide was determined by the sum of the peak areas from all identified charge states [66].

The 293T-derived Env samples were analyzed on a Q-Exactive mass spectrometer. De-glycosylated peptides were separated on a Dionex Ultimate 3000 RSLC nano system with a 75 μm × 15 cm Acclaim PepMap100 separating column protected by a 2-cm guard column. The flow rate was set at 300 nl/min. Buffer A and B were 3% ACN (0.1% FA) and 90% ACN (0.1% FA), respectively. A 130-minute gradient was run, consisting of the following steps: 0–10 min, 2–5% B; 10–90 min, 5–25% B; 90–112 min, 25–35% B; 112–115 min, 35–95%; 115–125 min, hold at 95% B; 125–129 min, 95–2% B; 129–130 min, hold at 2% B. Orbitrap MS1 spectra (AGC 3e6) were collected from 400–1,800 m/z at a resolution of 70 K followed by data dependent higher-energy collisional dissociation tandem mass spectrometry (HCD MS/MS) (resolution 35,000 and collision energy 31%) of the 12 most abundant ions using an isolation width of 1.4 Da. Dynamic exclusion time was set at 30s.

The PBMC-derived Env samples were analyzed on an Orbitrap Fusion Lumos tribrid mass spectrometer. Approximately 1 μg of de-glycosylated peptides were injected directly onto a 28 cm, 75 um ID column packed with 1.9 um Reprosil-Pur C18-AQ beads (Dr. Maisch GmbH). The flow rate was separated at 300 nl/min on an EASY-nLC 1200. Buffer A and B were 3% ACN (0.1% FA) and 90% ACN (0.1% FA), respectively. A 110-minute gradient was run, consisting of the following steps: 0–1 min, 2–6% B; 1–85 min, 6–30% B; 85–94 min, 30–60% B; 94–95 min, 60–90% B; 95–100 min, hold at 90% B; 100–101 min, 90–50% B; 101–110 min hold at 50% B. Peptides were eluted directly from the tip of the column and nanosprayed into the mass spectrometer. The Orbitrap Fusion Lumos was operated in a data-dependent mode. Full MS1 scans were collected in Orbitrap at 60 K resolution with a mass range of 350–2,000 m/z and an AGC target of 4e5. The cycle time was set to 2 s, and within this 2 s the most abundant ions per scan were selected for CID MS/MS in Orbitrap with an AGC target of 5e4. Maximum injection times were set to 50 ms for both MS and MS/MS scans. Monoisotopic precursor selection was enabled, and dynamic exclusion was used with exclusion duration of 45 s.

CMU06 Env trimer model was generated using a SOSIP.664 trimer (PDB 5FYJ) with all glycosites, except N674 due to lack of template. Glycans were added onto models based on the LC-MS/MS data of virion-associated Envs from 293T cells and PBMCs. For glycosites with high complex-type content, biantennary glycans with core fucose Gal2GlcNAc2Man3FucGlcNAc2 moieties were used. For those with high oligomannose content or undetected, Man5GlcNAc2 were added. Glycans that were undetected (e.g., N135, N234, N241, N262, N276, N289, N356, N386 and N625) are colored gray, while those unoccupied on PBMC-derived CMU06 are displayed by semi-transparent yellow patches.

### Statistical analysis

Comparison of SP swaps vs WT groups and controls was done using analysis of variance (ANOVA) or by Student’s t-test as mentioned in the figure legends. Statistical analyses were performed with GraphPad Prism 8.

## Supporting information

S1 Fig CMU06 gp160 sequence. N-glycosylation sites (red) are marked throughout the entire gp160 sequence that encompasses SP, constant regions (C1-C5), variable regions (V1-V5), and gp41.
(TIF)

S2 Fig Changes of neutralization sensitivity in the context of different Envs and SPs. AUC changes of SP-swapped vs WT for the different virus strains tested are shown for comparison. NT, not tested.
(TIF)

S3 Fig Glycan contents at glycosites on CMU06-WT (A), CMU06-398F1 (B), and CMU06-271.1 (C) produced in PBMCs vs 293T cells. Glycosites detected on at least one of the Envs from PBMCs- and 293T-derived viruses are shown. (D) Percentages of unoccupied, complex, and oligomannose glycans on total detected glycosites (gp120 and gp41) from viruses grown in 293T cells vs PBMCs. The number of total glycosites for each virus is shown in parentheses.
(TIF)

S4 Fig SP prediction. (A) SP prediction tool SignalP 3.0 applied to SPs of CMU06 WT and swap variants. Vertical red bars show the first amino acid after the cleavage site. (B) Probability of SP cleavage predicted by SignalP3.0. (C-D) PSIPRED-predicted MEMSAT-SVM helix orientation models (C) and DMPFold structures (D). One of the 5 structures predicted by PSIPRED for each SP is shown in panel D. α-helices: cyan, loops: magenta, residues C27, A29 and D31 around the cleavage site: magenta sticks.
(TIF)

## References

[1] Haeuptle MT, Flint N, Gough NM, Dobberstein B. A tripartite structure of the signals that determine protein insertion into the endoplasmic reticulum membrane. J Cell Biol. 1989 4;108(4):1227–36. 10.1083/jcb.108.4.1227 2784443 PMC2115504

[2] Checkley MA, Luttge BG, Freed EO. HIV-1 envelope glycoprotein biosynthesis, trafficking, and incorporation. Journal of molecular biology. 2011 7 22;410(4):582–608. 10.1016/j.jmb.2011.04.042 21762802 PMC3139147

[3] Land A, Braakman I. Folding of the human immunodeficiency virus type 1 envelope glycoprotein in the endoplasmic reticulum. Biochimie. 2001 8;83(8):783–90. 10.1016/s0300-9084(01)01314-1 11530211

[4] Li Y, Luo L, Rasool N, Kang CY. Glycosylation is necessary for the correct folding of human immunodeficiency virus gp120 in CD4 binding. Journal of virology. 1993 1;67(1):584–8. 10.1128/JVI.67.1.584-588.1993 8416385 PMC237399

[5] Julien JP, Cupo A, Sok D, Stanfield RL, Lyumkis D, Deller MC, et al Crystal structure of a soluble cleaved HIV-1 envelope trimer. Science. 2013 12 20;342(6165):1477–83. 10.1126/science.1245625 24179159 PMC3886632

[6] Lyumkis D, Julien JP, de Val N, Cupo A, Potter CS, Klasse PJ, et al Cryo-EM structure of a fully glycosylated soluble cleaved HIV-1 envelope trimer. Science. 2013 12 20;342(6165):1484–90. 10.1126/science.1245627 24179160 PMC3954647

[7] Pancera M, Zhou T, Druz A, Georgiev IS, Soto C, Gorman J, et al Structure and immune recognition of trimeric pre-fusion HIV-1 Env. Nature. 2014 10 23;514(7523):455–61. 10.1038/nature13808 25296255 PMC4348022

[8] Gottardo R, Bailer RT, Korber BT, Gnanakaran S, Phillips J, Shen X, et al Plasma IgG to linear epitopes in the V2 and V3 regions of HIV-1 gp120 correlate with a reduced risk of infection in the RV144 vaccine efficacy trial. PloS one. 2013;8(9):e75665 10.1371/journal.pone.0075665 24086607 PMC3784573

[9] Karasavvas N, Billings E, Rao M, Williams C, Zolla-Pazner S, Bailer RT, et al The Thai Phase III HIV Type 1 Vaccine trial (RV144) regimen induces antibodies that target conserved regions within the V2 loop of gp120. AIDS Res Hum Retroviruses. 2012 11;28(11):1444–57. 10.1089/aid.2012.0103 23035746 PMC3484815

[10] Zolla-Pazner S, Edlefsen PT, Rolland M, Kong XP, deCamp A, Gottardo R, et al Vaccine-induced Human Antibodies Specific for the Third Variable Region of HIV-1 gp120 Impose Immune Pressure on Infecting Viruses. EBioMedicine. 2014 11 01;1(1):37–45. 10.1016/j.ebiom.2014.10.022 25599085 PMC4293639

[11] Upadhyay C, Mayr LM, Zhang J, Kumar R, Gorny MK, Nadas A, et al Distinct mechanisms regulate exposure of neutralizing epitopes in the V2 and V3 loops of HIV-1 envelope. Journal of virology. 2014 11;88(21):12853–65. 10.1128/JVI.02125-14 25165106 PMC4248937

[12] Moore PL, Gray ES, Wibmer CK, Bhiman JN, Nonyane M, Sheward DJ, et al Evolution of an HIV glycan-dependent broadly neutralizing antibody epitope through immune escape. Nature medicine. 2012 11;18(11):1688–92. 10.1038/nm.2985 23086475 PMC3494733

[13] Lynch RM, Wong P, Tran L, O'Dell S, Nason MC, Li Y, et al HIV-1 fitness cost associated with escape from the VRC01 class of CD4 binding site neutralizing antibodies. Journal of virology. 2015 4;89(8):4201–13. 10.1128/JVI.03608-14 25631091 PMC4442379

[14] Wei X, Decker JM, Wang S, Hui H, Kappes JC, Wu X, et al Antibody neutralization and escape by HIV-1. Nature. 2003 3 20;422(6929):307–12. 10.1038/nature01470 12646921

[15] Doria-Rose NA, Schramm CA, Gorman J, Moore PL, Bhiman JN, DeKosky BJ, et al Developmental pathway for potent V1V2-directed HIV-neutralizing antibodies. Nature. 2014 5 1;509(7498):55–62. 10.1038/nature13036 24590074 PMC4395007

[16] McLellan JS, Pancera M, Carrico C, Gorman J, Julien JP, Khayat R, et al Structure of HIV-1 gp120 V1/V2 domain with broadly neutralizing antibody PG9. Nature. 2011 12 15;480(7377):336–43. 10.1038/nature10696 22113616 PMC3406929

[17] Pancera M, Shahzad-Ul-Hussan S, Doria-Rose NA, McLellan JS, Bailer RT, Dai K, et al Structural basis for diverse N-glycan recognition by HIV-1-neutralizing V1-V2-directed antibody PG16. Nature structural & molecular biology. 2013 7;20(7):804–13. 10.1038/nsmb.2600 23708607 PMC4046252

[18] Pejchal R, Doores KJ, Walker LM, Khayat R, Huang PS, Wang SK, et al A potent and broad neutralizing antibody recognizes and penetrates the HIV glycan shield. Science. 2011 11 25;334(6059):1097–103. 10.1126/science.1213256 21998254 PMC3280215

[19] Pan R, Gorny MK, Zolla-Pazner S, Kong XP. The V1V2 Region of HIV-1 gp120 Forms a Five-Stranded Beta Barrel. Journal of virology. 2015 8;89(15):8003–10. 10.1128/JVI.00754-15 26018158 PMC4505664

[20] Spurrier B, Sampson J, Gorny MK, Zolla-Pazner S, Kong XP. Functional implications of the binding mode of a human conformation-dependent V2 monoclonal antibody against HIV. Journal of virology. 2014 4;88(8):4100–12. 10.1128/JVI.03153-13 24478429 PMC3993739

[21] Liao HX, Bonsignori M, Alam SM, McLellan JS, Tomaras GD, Moody MA, et al Vaccine induction of antibodies against a structurally heterogeneous site of immune pressure within HIV-1 envelope protein variable regions 1 and 2. Immunity. 2013 1 24;38(1):176–86. 10.1016/j.immuni.2012.11.011 23313589 PMC3569735

[22] van Eeden C, Wibmer CK, Scheepers C, Richardson SI, Nonyane M, Lambson B, et al V2-Directed Vaccine-like Antibodies from HIV-1 Infection Identify an Additional K169-Binding Light Chain Motif with Broad ADCC Activity. Cell Rep. 2018 12 11;25(11):3123–35 e6. 10.1016/j.celrep.2018.11.058 30540944 PMC6342559

[23] Gorny MK, Pan R, Williams C, Wang XH, Volsky B, O'Neal T, et al Functional and immunochemical cross-reactivity of V2-specific monoclonal antibodies from HIV-1-infected individuals. Virology. 2012 6 5;427(2):198–207. 10.1016/j.virol.2012.02.003 22402248 PMC3572902

[24] Mayr LM, Cohen S, Spurrier B, Kong XP, Zolla-Pazner S. Epitope mapping of conformational V2-specific anti-HIV human monoclonal antibodies reveals an immunodominant site in V2. PloS one. 2013;8(7):e70859 10.1371/journal.pone.0070859 23923028 PMC3726596

[25] Bonsignori M, Hwang KK, Chen X, Tsao CY, Morris L, Gray E, et al Analysis of a clonal lineage of HIV-1 envelope V2/V3 conformational epitope-specific broadly neutralizing antibodies and their inferred unmutated common ancestors. Journal of virology. 2011 10;85(19):9998–10009. 10.1128/JVI.05045-11 21795340 PMC3196428

[26] Walker LM, Phogat SK, Chan-Hui PY, Wagner D, Phung P, Goss JL, et al Broad and potent neutralizing antibodies from an African donor reveal a new HIV-1 vaccine target. Science. 2009 10 9;326(5950):285–9. 10.1126/science.1178746 19729618 PMC3335270

[27] Upadhyay C, Feyznezhad R, Yang W, Zhang H, Zolla-Pazner S, Hioe CE. Alterations of HIV-1 envelope phenotype and antibody-mediated neutralization by signal peptide mutations. PLoS pathogens. 2018 1 25;14(1):e1006812 10.1371/journal.ppat.1006812 29370305 PMC5800646

[28] Yolitz J, Schwing C, Chang J, Van Ryk D, Nawaz F, Wei D, et al Signal peptide of HIV envelope protein impacts glycosylation and antigenicity of gp120. Proceedings of the National Academy of Sciences of the United States of America. 2018 3 6;115(10):2443–8. 10.1073/pnas.1722627115 29463753 PMC5877976

[29] da Silva JX, Franco OL, Lemos MA, Gondim MV, Prosdocimi F, Arganaraz ER. Sequence variations of Env signal peptide alleles in different clinical stages of HIV infection. Peptides. 2011 9;32(9):1800–6. 10.1016/j.peptides.2011.07.014 21816188

[30] Asmal M, Hellmann I, Liu W, Keele BF, Perelson AS, Bhattacharya T, et al A signature in HIV-1 envelope leader peptide associated with transition from acute to chronic infection impacts envelope processing and infectivity. PloS one. 2011;6(8):e23673 10.1371/journal.pone.0023673 21876761 PMC3158090

[31] Li Y, Luo L, Thomas DY, Kang CY. Control of expression, glycosylation, and secretion of HIV-1 gp120 by homologous and heterologous signal sequences. Virology. 1994 10;204(1):266–78. 10.1006/viro.1994.1531 8091657

[32] AlSalmi W, Mahalingam M, Ananthaswamy N, Hamlin C, Flores D, Gao G, et al A New Approach to Produce HIV-1 Envelope Trimers: BOTH CLEAVAGE AND PROPER GLYCOSYLATION ARE ESSENTIAL TO GENERATE AUTHENTIC TRIMERS. J Biol Chem. 2015 8 7;290(32):19780–95. 10.1074/jbc.M115.656611 26088135 PMC4528139

[33] Sanders RW, Derking R, Cupo A, Julien JP, Yasmeen A, de Val N, et al A next-generation cleaved, soluble HIV-1 Env trimer, BG505 SOSIP.664 gp140, expresses multiple epitopes for broadly neutralizing but not non-neutralizing antibodies. PLoS pathogens. 2013 9;9(9):e1003618 10.1371/journal.ppat.1003618 24068931 PMC3777863

[34] Alam SM, Liao HX, Tomaras GD, Bonsignori M, Tsao CY, Hwang KK, et al Antigenicity and immunogenicity of RV144 vaccine AIDSVAX clade E envelope immunogen is enhanced by a gp120 N-terminal deletion. Journal of virology. 2013 2;87(3):1554–68. 10.1128/JVI.00718-12 23175357 PMC3554162

[35] Lasky LA, Groopman JE, Fennie CW, Benz PM, Capon DJ, Dowbenko DJ, et al Neutralization of the AIDS retrovirus by antibodies to a recombinant envelope glycoprotein. Science. 1986 7 11;233(4760):209–12. 10.1126/science.3014647 3014647

[36] Berman PW. Development of bivalent rgp120 vaccines to prevent HIV type 1 infection. AIDS Res Hum Retroviruses. 1998 10;14 Suppl 3:S277–89. 9814956

[37] Li Y, Bergeron JJ, Luo L, Ou WJ, Thomas DY, Kang CY. Effects of inefficient cleavage of the signal sequence of HIV-1 gp 120 on its association with calnexin, folding, and intracellular transport. Proceedings of the National Academy of Sciences of the United States of America. 1996 9 3;93(18):9606–11. 10.1073/pnas.93.18.9606 8790377 PMC38475

[38] Snapp EL, McCaul N, Quandte M, Cabartova Z, Bontjer I, Kallgren C, et al Structure and topology around the cleavage site regulate post-translational cleavage of the HIV-1 gp160 signal peptide. eLife. 2017 7 28;6 10.7554/eLife.26067 28753126 PMC5577925

[39] Land A, Zonneveld D, Braakman I. Folding of HIV-1 envelope glycoprotein involves extensive isomerization of disulfide bonds and conformation-dependent leader peptide cleavage. FASEB J. 2003 6;17(9):1058–67. 10.1096/fj.02-0811com 12773488

[40] von Heijne G. Signal sequences. The limits of variation. Journal of molecular biology. 1985 7 5;184(1):99–105. 10.1016/0022-2836(85)90046-4 4032478

[41] von Heijne G, Gavel Y. Topogenic signals in integral membrane proteins. Eur J Biochem. 1988 7 1;174(4):671–8. 10.1111/j.1432-1033.1988.tb14150.x 3134198

[42] Arnold A, Horst SA, Gardella TJ, Baba H, Levine MA, Kronenberg HM. Mutation of the signal peptide-encoding region of the preproparathyroid hormone gene in familial isolated hypoparathyroidism. J Clin Invest. 1990 10;86(4):1084–7. 10.1172/JCI114811 2212001 PMC296835

[43] Schiemann WP, Rotzer D, Pfeifer WM, Levi E, Rai KR, Knaus P, et al Transforming growth factor-beta (TGF-beta)-resistant B cells from chronic lymphocytic leukemia patients contain recurrent mutations in the signal sequence of the type I TGF-beta receptor. Cancer Detect Prev. 2004;28(1):57–64. 10.1016/j.cdp.2003.11.001 15041079

[44] Ding B, Kull B, Liu Z, Mottagui-Tabar S, Thonberg H, Gu HF, et al Human neuropeptide Y signal peptide gain-of-function polymorphism is associated with increased body mass index: possible mode of function. Regul Pept. 2005 4 15;127(1–3):45–53. 10.1016/j.regpep.2004.10.011 15680469

[45] Meur G, Simon A, Harun N, Virally M, Dechaume A, Bonnefond A, et al Insulin gene mutations resulting in early-onset diabetes: marked differences in clinical presentation, metabolic status, and pathogenic effect through endoplasmic reticulum retention. Diabetes. 2010 3;59(3):653–61. 10.2337/db09-1091 20007936 PMC2828668

[46] Piersma D, Berns EM, Verhoef-Post M, Uitterlinden AG, Braakman I, Pols HA, et al A common polymorphism renders the luteinizing hormone receptor protein more active by improving signal peptide function and predicts adverse outcome in breast cancer patients. J Clin Endocrinol Metab. 2006 4;91(4):1470–6. 10.1210/jc.2005-2156 16464948

[47] Witt H, Luck W, Becker M. A signal peptide cleavage site mutation in the cationic trypsinogen gene is strongly associated with chronic pancreatitis. Gastroenterology. 1999 7;117(1):7–10. 10.1016/s0016-5085(99)70543-3 10381903

[48] Lindemann D, Pietschmann T, Picard-Maureau M, Berg A, Heinkelein M, Thurow J, et al A particle-associated glycoprotein signal peptide essential for virus maturation and infectivity. Journal of virology. 2001 7;75(13):5762–71. 10.1128/JVI.75.13.5762-5771.2001 11390578 PMC114292

[49] Feldman D, Roniger M, Bar-Sinai A, Braitbard O, Natan C, Love DC, et al The signal peptide of mouse mammary tumor virus-env: a phosphoprotein tumor modulator. Mol Cancer Res. 2012 8;10(8):1077–86. 10.1158/1541-7786.MCR-11-0581 22740636

[50] Seaman MS, Janes H, Hawkins N, Grandpre LE, Devoy C, Giri A, et al Tiered categorization of a diverse panel of HIV-1 Env pseudoviruses for assessment of neutralizing antibodies. Journal of virology. 2010 2;84(3):1439–52. 10.1128/JVI.02108-09 19939925 PMC2812321

[51] deCamp A, Hraber P, Bailer RT, Seaman MS, Ochsenbauer C, Kappes J, et al Global panel of HIV-1 Env reference strains for standardized assessments of vaccine-elicited neutralizing antibodies. Journal of virology. 2014 3;88(5):2489–507. 10.1128/JVI.02853-13 24352443 PMC3958090

[52] Olausson J, Tibell L, Jonsson BH, Pahlsson P. Detection of a high affinity binding site in recombinant Aleuria aurantia lectin. Glycoconj J. 2008 11;25(8):753–62. 10.1007/s10719-008-9135-7 18493851

[53] Hester G, Kaku H, Goldstein IJ, Wright CS. Structure of mannose-specific snowdrop (Galanthus nivalis) lectin is representative of a new plant lectin family. Nat Struct Biol. 1995 6;2(6):472–9. 10.1038/nsb0695-472 7664110

[54] Moore PL, Crooks ET, Porter L, Zhu P, Cayanan CS, Grise H, et al Nature of nonfunctional envelope proteins on the surface of human immunodeficiency virus type 1. Journal of virology. 2006 3;80(5):2515–28. 10.1128/JVI.80.5.2515-2528.2006 16474158 PMC1395414

[55] Crooks ET, Tong T, Osawa K, Binley JM. Enzyme digests eliminate nonfunctional Env from HIV-1 particle surfaces, leaving native Env trimers intact and viral infectivity unaffected. Journal of virology. 2011 6;85(12):5825–39. 10.1128/JVI.00154-11 21471242 PMC3126298

[56] Sok D, Pauthner M, Briney B, Lee JH, Saye-Francisco KL, Hsueh J, et al A Prominent Site of Antibody Vulnerability on HIV Envelope Incorporates a Motif Associated with CCR5 Binding and Its Camouflaging Glycans. Immunity. 2016 7 19;45(1):31–45. 10.1016/j.immuni.2016.06.026 27438765 PMC4990068

[57] Rantalainen K, Berndsen ZT, Murrell S, Cao L, Omorodion O, Torres JL, et al Co-evolution of HIV Envelope and Apex-Targeting Neutralizing Antibody Lineage Provides Benchmarks for Vaccine Design. Cell Rep. 2018 6 12;23(11):3249–61. 10.1016/j.celrep.2018.05.046 29898396 PMC6019700

[58] Doores KJ, Burton DR. Variable loop glycan dependency of the broad and potent HIV-1-neutralizing antibodies PG9 and PG16. Journal of virology. 2010 10;84(20):10510–21. 10.1128/JVI.00552-10 20686044 PMC2950566

[59] Lee JH, Ozorowski G, Ward AB. Cryo-EM structure of a native, fully glycosylated, cleaved HIV-1 envelope trimer. Science. 2016 3 4;351(6277):1043–8. 10.1126/science.aad2450 26941313 PMC5001164

[60] Dubrovskaya V, Tran K, Ozorowski G, Guenaga J, Wilson R, Bale S, et al Vaccination with Glycan-Modified HIV NFL Envelope Trimer-Liposomes Elicits Broadly Neutralizing Antibodies to Multiple Sites of Vulnerability. Immunity. 2019 11 19;51(5):915–29 e7. 10.1016/j.immuni.2019.10.008 31732167 PMC6891888

[61] Blattner C, Lee JH, Sliepen K, Derking R, Falkowska E, de la Pena AT, et al Structural delineation of a quaternary, cleavage-dependent epitope at the gp41-gp120 interface on intact HIV-1 Env trimers. Immunity. 2014 5 15;40(5):669–80. 10.1016/j.immuni.2014.04.008 24768348 PMC4057017

[62] Falkowska E, Le KM, Ramos A, Doores KJ, Lee JH, Blattner C, et al Broadly neutralizing HIV antibodies define a glycan-dependent epitope on the prefusion conformation of gp41 on cleaved envelope trimers. Immunity. 2014 5 15;40(5):657–68. 10.1016/j.immuni.2014.04.009 24768347 PMC4070425

[63] Huang J, Kang BH, Pancera M, Lee JH, Tong T, Feng Y, et al Broad and potent HIV-1 neutralization by a human antibody that binds the gp41-gp120 interface. Nature. 2014 9 3 10.1038/nature13601 25186731 PMC4224615

[64] Go EP, Liao HX, Alam SM, Hua D, Haynes BF, Desaire H. Characterization of host-cell line specific glycosylation profiles of early transmitted/founder HIV-1 gp120 envelope proteins. J Proteome Res. 2013 3 1;12(3):1223–34. 10.1021/pr300870t 23339644 PMC3674872

[65] Raska M, Takahashi K, Czernekova L, Zachova K, Hall S, Moldoveanu Z, et al Glycosylation patterns of HIV-1 gp120 depend on the type of expressing cells and affect antibody recognition. J Biol Chem. 2010 7 2;285(27):20860–9. 10.1074/jbc.M109.085472 20439465 PMC2898351

[66] Behrens AJ, Vasiljevic S, Pritchard LK, Harvey DJ, Andev RS, Krumm SA, et al Composition and Antigenic Effects of Individual Glycan Sites of a Trimeric HIV-1 Envelope Glycoprotein. Cell Rep. 2016 3 22;14(11):2695–706. 10.1016/j.celrep.2016.02.058 26972002 PMC4805854

[67] Shivatare VS, Shivatare SS, Lee CD, Liang CH, Liao KS, Cheng YY, et al Unprecedented Role of Hybrid N-Glycans as Ligands for HIV-1 Broadly Neutralizing Antibodies. J Am Chem Soc. 2018 4 18;140(15):5202–10. 10.1021/jacs.8b00896 29578688

[68] O'Rourke SM, Sutthent R, Phung P, Mesa KA, Frigon NL, To B, et al Glycans flanking the hypervariable connecting peptide between the A and B strands of the V1/V2 domain of HIV-1 gp120 confer resistance to antibodies that neutralize CRF01_AE viruses. PloS one. 2015;10(3):e0119608 10.1371/journal.pone.0119608 25793890 PMC4368187

[69] Behrens AJ, Harvey DJ, Milne E, Cupo A, Kumar A, Zitzmann N, et al Molecular Architecture of the Cleavage-Dependent Mannose Patch on a Soluble HIV-1 Envelope Glycoprotein Trimer. Journal of virology. 2017 1 15;91(2). 10.1128/JVI.01894-16 27807235 PMC5215339

[70] Pritchard LK, Vasiljevic S, Ozorowski G, Seabright GE, Cupo A, Ringe R, et al Structural Constraints Determine the Glycosylation of HIV-1 Envelope Trimers. Cell Rep. 2015 6 16;11(10):1604–13. 10.1016/j.celrep.2015.05.017 26051934 PMC4555872

[71] Struwe WB, Chertova E, Allen JD, Seabright GE, Watanabe Y, Harvey DJ, et al Site-Specific Glycosylation of Virion-Derived HIV-1 Env Is Mimicked by a Soluble Trimeric Immunogen. Cell Rep. 2018 8 21;24(8):1958–66 e5. 10.1016/j.celrep.2018.07.080 30134158 PMC6113929

[72] Ringe RP, Ozorowski G, Yasmeen A, Cupo A, Cruz Portillo VM, Pugach P, et al Improving the Expression and Purification of Soluble, Recombinant Native-Like HIV-1 Envelope Glycoprotein Trimers by Targeted Sequence Changes. Journal of virology. 2017 6 15;91(12). 10.1128/JVI.00264-17 28381572 PMC5446630

[73] Han Q, Jones JA, Nicely NI, Reed RK, Shen X, Mansouri K, et al Difficult-to-neutralize global HIV-isolates are neutralized by antibodies targeting open envelope conformations. Nat Commun. 2019 7 1;10(1):2898 10.1038/s41467-019-10899-2 31263112 PMC6602974

[74] Zolla-Pazner S, Cohen SS, Boyd D, Kong XP, Seaman M, Nussenzweig M, et al Structure/Function Studies Involving the V3 Region of the HIV-1 Envelope Delineate Multiple Factors That Affect Neutralization Sensitivity. Journal of virology. 2016 1;90(2):636–49. 10.1128/JVI.01645-15 26491157 PMC4702699

[75] de Taeye SW, de la Pena AT, Vecchione A, Scutigliani E, Sliepen K, Burger JA, et al Stabilization of the gp120 V3 loop through hydrophobic interactions reduces the immunodominant V3-directed non-neutralizing response to HIV-1 envelope trimers. J Biol Chem. 2018 2 2;293(5):1688–701. 10.1074/jbc.RA117.000709 29222332 PMC5798299

[76] Hogan MJ, Conde-Motter A, Jordan APO, Yang L, Cleveland B, Guo W, et al Increased surface expression of HIV-1 envelope is associated with improved antibody response in vaccinia prime/protein boost immunization. Virology. 2018 1 15;514:106–17. 10.1016/j.virol.2017.10.013 29175625 PMC5770335

[77] Rutkowski DT, Ott CM, Polansky JR, Lingappa VR. Signal sequences initiate the pathway of maturation in the endoplasmic reticulum lumen. J Biol Chem. 2003 8 8;278(32):30365–72. 10.1074/jbc.M302117200 12771148

[78] Martoglio B, Dobberstein B. Signal sequences: more than just greasy peptides. Trends in cell biology. 1998 10;8(10):410–5. 10.1016/s0962-8924(98)01360-9 9789330

[79] Nielsen H, Engelbrecht J, Brunak S, von Heijne G. Identification of prokaryotic and eukaryotic signal peptides and prediction of their cleavage sites. Protein Eng. 1997 1;10(1):1–6. 10.1093/protein/10.1.1 9051728

[80] Buchan DW, Ward SM, Lobley AE, Nugent TC, Bryson K, Jones DT. Protein annotation and modelling servers at University College London. Nucleic Acids Res. 2010 7;38(Web Server issue):W563–8. 10.1093/nar/gkq427 20507913 PMC2896093

[81] Buchan DWA, Jones DT. The PSIPRED Protein Analysis Workbench: 20 years on. Nucleic Acids Res. 2019 7 2;47(W1):W402–W7. 10.1093/nar/gkz297 31251384 PMC6602445

[82] Wang JY, Song WT, Li Y, Chen WJ, Yang D, Zhong GC, et al Improved expression of secretory and trimeric proteins in mammalian cells via the introduction of a new trimer motif and a mutant of the tPA signal sequence. Appl Microbiol Biotechnol. 2011 8;91(3):731–40. 10.1007/s00253-011-3297-0 21556920

[83] Nilsson I, Lara P, Hessa T, Johnson AE, von Heijne G, Karamyshev AL. The code for directing proteins for translocation across ER membrane: SRP cotranslationally recognizes specific features of a signal sequence. Journal of molecular biology. 2015 3 27;427(6 Pt A):1191–201. 24979680 10.1016/j.jmb.2014.06.014 PMC4277940

[84] Peterson JH, Woolhead CA, Bernstein HD. Basic amino acids in a distinct subset of signal peptides promote interaction with the signal recognition particle. J Biol Chem. 2003 11 14;278(46):46155–62. 10.1074/jbc.M309082200 12949068

[85] Walter P, Ibrahimi I, Blobel G. Translocation of proteins across the endoplasmic reticulum. I. Signal recognition protein (SRP) binds to in-vitro-assembled polysomes synthesizing secretory protein. J Cell Biol. 1981 11;91(2 Pt 1):545–50. 10.1083/jcb.91.2.545 7309795 PMC2111968

[86] Earl PL, Moss B, Doms RW. Folding, interaction with GRP78-BiP, assembly, and transport of the human immunodeficiency virus type 1 envelope protein. Journal of virology. 1991 4;65(4):2047–55. 10.1128/JVI.65.4.2047-2055.1991 1900540 PMC240054

[87] Zheng N, Gierasch LM. Signal sequences: the same yet different. Cell. 1996 9 20;86(6):849–52. 10.1016/s0092-8674(00)80159-2 8808619

[88] Adams H, Scotti PA, De Cock H, Luirink J, Tommassen J. The presence of a helix breaker in the hydrophobic core of signal sequences of secretory proteins prevents recognition by the signal-recognition particle in Escherichia coli. Eur J Biochem. 2002 11;269(22):5564–71. 10.1046/j.1432-1033.2002.03262.x 12423355

[89] Keenan RJ, Freymann DM, Walter P, Stroud RM. Crystal structure of the signal sequence binding subunit of the signal recognition particle. Cell. 1998 7 24;94(2):181–91. 10.1016/s0092-8674(00)81418-x 9695947

[90] Martoglio B, Graf R, Dobberstein B. Signal peptide fragments of preprolactin and HIV-1 p-gp160 interact with calmodulin. EMBO J. 1997 11 17;16(22):6636–45. 10.1093/emboj/16.22.6636 9362478 PMC1170268

[91] Li M, Gao F, Mascola JR, Stamatatos L, Polonis VR, Koutsoukos M, et al Human immunodeficiency virus type 1 env clones from acute and early subtype B infections for standardized assessments of vaccine-elicited neutralizing antibodies. Journal of virology. 2005 8;79(16):10108–25. 10.1128/JVI.79.16.10108-10125.2005 16051804 PMC1182643

[92] Gorny MK, Moore JP, Conley AJ, Karwowska S, Sodroski J, Williams C, et al Human anti-V2 monoclonal antibody that neutralizes primary but not laboratory isolates of human immunodeficiency virus type 1. Journal of virology. 1994 12;68(12):8312–20. 10.1128/JVI.68.12.8312-8320.1994 7525987 PMC237300

[93] Nyambi PN, Mbah HA, Burda S, Williams C, Gorny MK, Nadas A, et al Conserved and exposed epitopes on intact, native, primary human immunodeficiency virus type 1 virions of group M. Journal of virology. 2000 8;74(15):7096–107. 10.1128/jvi.74.15.7096-7107.2000 10888650 PMC112228

[94] Pinter A, Honnen WJ, He Y, Gorny MK, Zolla-Pazner S, Kayman SC. The V1/V2 domain of gp120 is a global regulator of the sensitivity of primary human immunodeficiency virus type 1 isolates to neutralization by antibodies commonly induced upon infection. Journal of virology. 2004 5;78(10):5205–15. 10.1128/jvi.78.10.5205-5215.2004 15113902 PMC400352

[95] Gorny MK, Conley AJ, Karwowska S, Buchbinder A, Xu JY, Emini EA, et al Neutralization of diverse human immunodeficiency virus type 1 variants by an anti-V3 human monoclonal antibody. Journal of virology. 1992 12;66(12):7538–42. 10.1128/JVI.66.12.7538-7542.1992 1433529 PMC240465

[96] Gorny MK, Williams C, Volsky B, Revesz K, Wang XH, Burda S, et al Cross-clade neutralizing activity of human anti-V3 monoclonal antibodies derived from the cells of individuals infected with non-B clades of human immunodeficiency virus type 1. Journal of virology. 2006 7;80(14):6865–72. 10.1128/JVI.02202-05 16809292 PMC1489067

[97] Hioe CE, Wrin T, Seaman MS, Yu X, Wood B, Self S, et al Anti-V3 monoclonal antibodies display broad neutralizing activities against multiple HIV-1 subtypes. PloS one. 2010;5(4):e10254 10.1371/journal.pone.0010254 20421997 PMC2858080

[98] Gigler A, Dorsch S, Hemauer A, Williams C, Kim S, Young NS, et al Generation of neutralizing human monoclonal antibodies against parvovirus B19 proteins. Journal of virology. 1999 3;73(3):1974–9. 10.1128/JVI.73.3.1974-1979.1999 9971777 PMC104439

[99] Wu X, Yang ZY, Li Y, Hogerkorp CM, Schief WR, Seaman MS, et al Rational design of envelope identifies broadly neutralizing human monoclonal antibodies to HIV-1. Science. 2010 8 13;329(5993):856–61. 10.1126/science.1187659 20616233 PMC2965066

[100] Barbas CF 3rd, Bjorling E, Chiodi F, Dunlop N, Cababa D, Jones TM, et al Recombinant human Fab fragments neutralize human type 1 immunodeficiency virus in vitro. Proceedings of the National Academy of Sciences of the United States of America. 1992 10 1;89(19):9339–43. 10.1073/pnas.89.19.9339 1384050 PMC50122

[101] Scheid JF, Mouquet H, Ueberheide B, Diskin R, Klein F, Oliveira TY, et al Sequence and structural convergence of broad and potent HIV antibodies that mimic CD4 binding. Science. 2011 9 16;333(6049):1633–7. 10.1126/science.1207227 21764753 PMC3351836

[102] Thali M, Moore JP, Furman C, Charles M, Ho DD, Robinson J, et al Characterization of conserved human immunodeficiency virus type 1 gp120 neutralization epitopes exposed upon gp120-CD4 binding. Journal of virology. 1993 7;67(7):3978–88. 10.1128/JVI.67.7.3978-3988.1993 7685405 PMC237765

[103] Buchacher A, Predl R, Strutzenberger K, Steinfellner W, Trkola A, Purtscher M, et al Generation of human monoclonal antibodies against HIV-1 proteins; electrofusion and Epstein-Barr virus transformation for peripheral blood lymphocyte immortalization. AIDS Res Hum Retroviruses. 1994 4;10(4):359–69. 10.1089/aid.1994.10.359 7520721

[104] Montefiori DC. Evaluating neutralizing antibodies against HIV, SIV, and SHIV in luciferase reporter gene assays. Current protocols in immunology. 2005 1;Chapter 12:Unit 12 1. 10.1002/0471142735.im1211s64 18432938

[105] Altman JB, Liu X, Itri V, Zolla-Pazner S, Powell RLR. Optimized protocol for detection of native, full-length HIV-1 envelope on the surface of transfected cells. Health Sci Rep. 2018 9;1(9):e74 10.1002/hsr2.74 30623097 PMC6266377

[106] Cao L, Diedrich JK, Kulp DW, Pauthner M, He L, Park SR, et al Global site-specific N-glycosylation analysis of HIV envelope glycoprotein. Nat Commun. 2017 3 28;8:14954 10.1038/ncomms14954 28348411 PMC5379070

[107] Angel PM, Lim JM, Wells L, Bergmann C, Orlando R. A potential pitfall in 18O-based N-linked glycosylation site mapping. Rapid Commun Mass Spectrom. 2007;21(5):674–82. 10.1002/rcm.2874 17279607

[108] Kaji H, Saito H, Yamauchi Y, Shinkawa T, Taoka M, Hirabayashi J, et al Lectin affinity capture, isotope-coded tagging and mass spectrometry to identify N-linked glycoproteins. Nature biotechnology. 2003 6;21(6):667–72. 10.1038/nbt829 12754521

