# Supplementary figures and images for: Signal peptide of HIV-1 envelope modulates glycosylation impacting exposure of V1V2 and other epitopes
Source: PLoS Pathog. 2020 Dec 28;16(12):e1009185. doi: 10.1371/journal.ppat.1009185 (PMC7793277; doi:10.1371/journal.ppat.1009185)

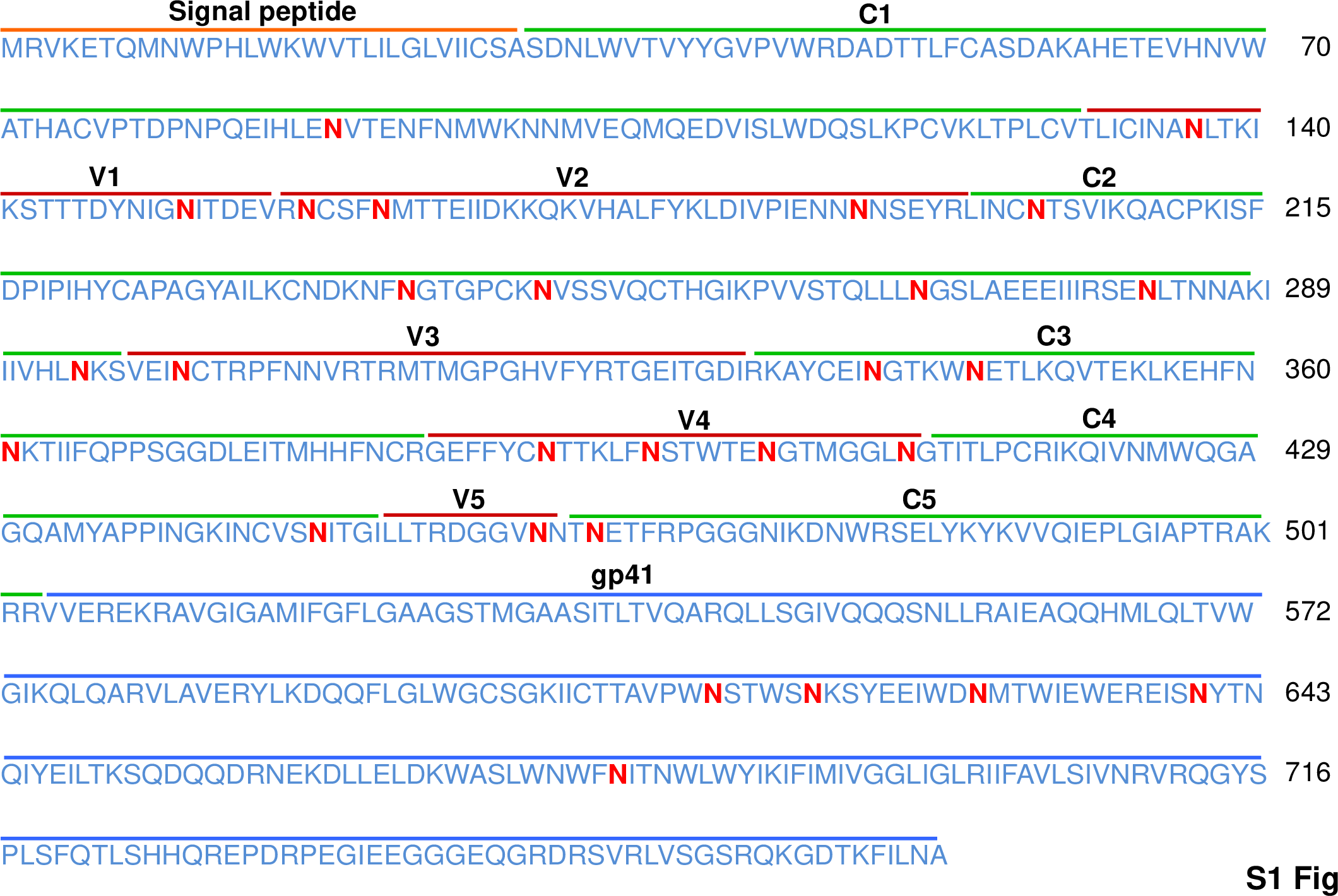

Supplement: S1 Fig — N-glycosylation sites (red) are marked throughout the entire gp160 sequence that encompasses SP, constant regions (C1-C5), variable regions (V1-V5), and gp41. (TIF) [file ppat.1009185.s001.tif]

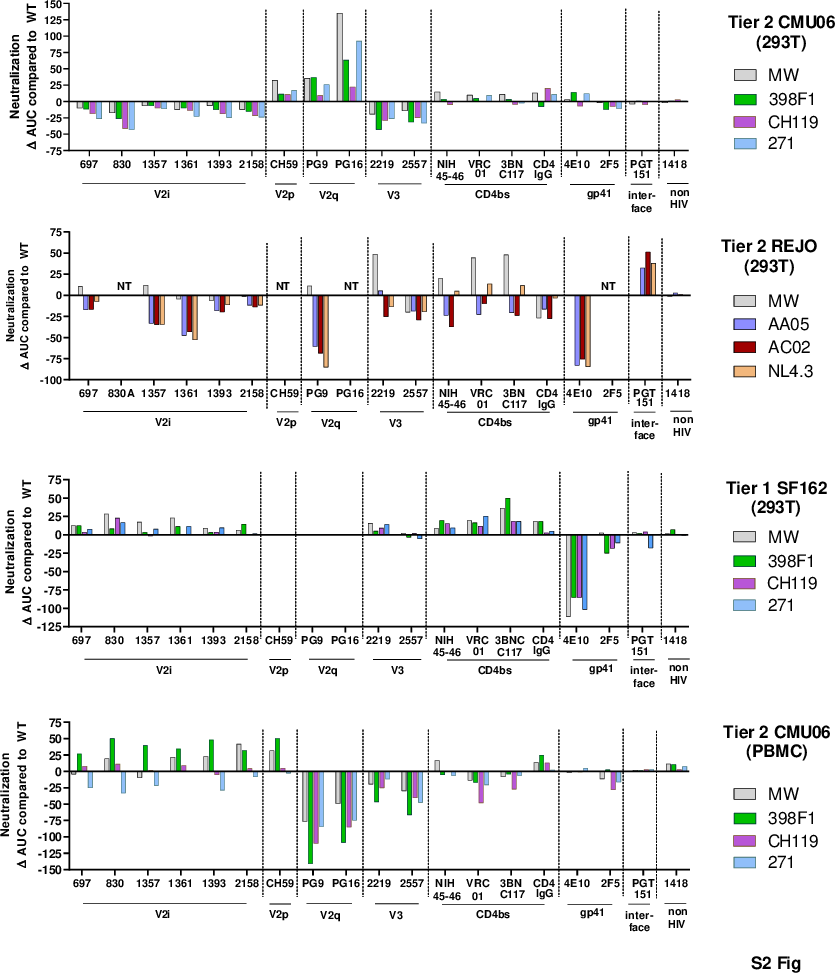

Supplement: S2 Fig — AUC changes of SP-swapped vs WT for the different virus strains tested are shown for comparison. NT, not tested. (TIF) [file ppat.1009185.s002.tif]

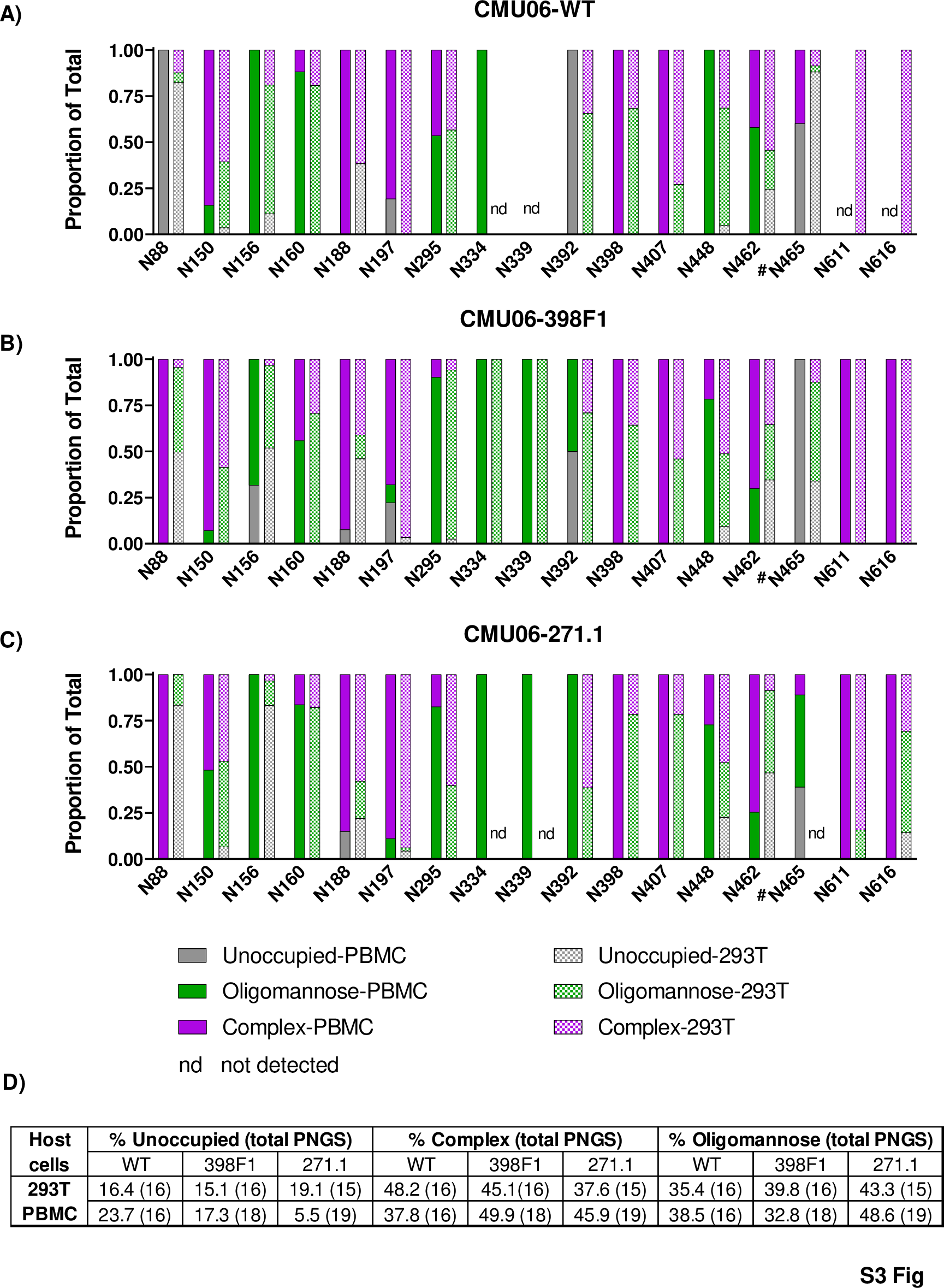

Supplement: S3 Fig — Glycosites detected on at least one of the Envs from PBMCs- and 293T-derived viruses are shown. (D) Percentages of unoccupied, complex, and oligomannose glycans on total detected glycosites (gp120 and gp41) from viruses grown in 293T cells vs PBMCs. The number of total glycosites for each virus is shown in parentheses. (TIF) [file ppat.1009185.s003.tif]

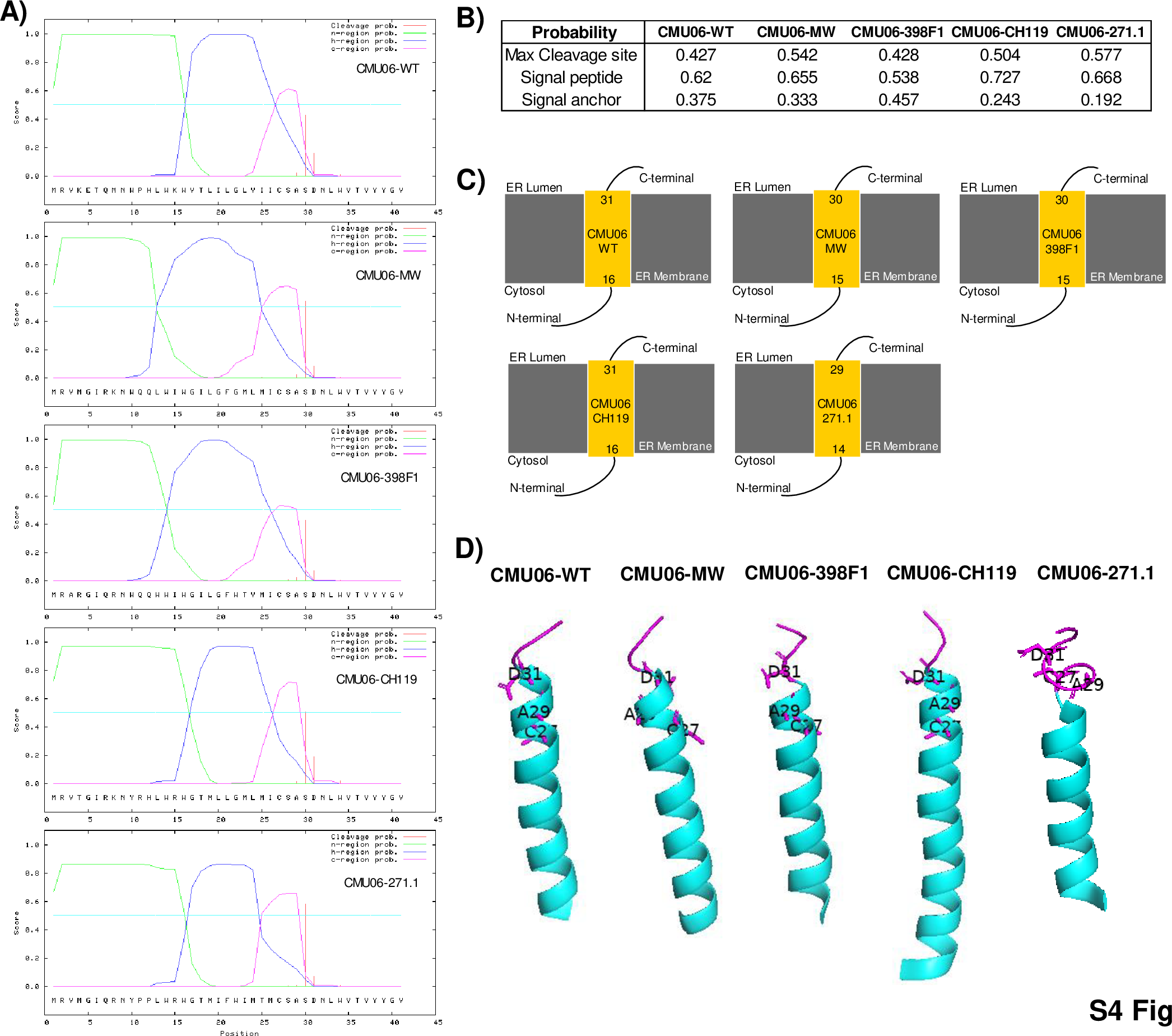

Supplement: S4 Fig — (A) SP prediction tool SignalP 3.0 applied to SPs of CMU06 WT and swap variants. Vertical red bars show the first amino acid after the cleavage site. (B) Probability of SP cleavage predicted by SignalP3.0. (C-D) PSIPRED-predicted MEMSAT-SVM helix orientation models (C) and DMPFold structures (D). One of the 5 structures predicted by PSIPRED for each SP is shown in panel D. α-helices: cyan, loops: magenta, residues C27, A29 and D31 around the cleavage site: magenta sticks. (TIF) [file ppat.1009185.s004.tif]
